# Multi-method approaches to develop and refine a tobacco cessation workflow within a federally qualified health center

**DOI:** 10.1186/s43058-026-01038-2

**Published:** 2026-07-06

**Authors:** Linda Salgin, Jerel P. Calzo, Jennifer K. Felner, David Strong, Marva Seifert, Borsika A. Rabin

**Affiliations:** 1 The Herbert Wertheim School of Public Health and Human Longevity Science, UC San Diego, 9500 Gilman Dr, La Jolla, San Diego, CA 92093 USA; 2 San Ysidro Health, 1601 Precision Park Lane, San Ysidro, CA 92173 USA; 3 School of Public Health, San Diego State University, San Diego, CA USA; 4 Division of Pulmonary, School of Medicine, Critical Care, and Sleep Medicine, UC San Diego, 9500 Gilman Dr, La Jolla, San Diego, CA 92093 USA; 5 Dissemination and Implementation Science Center, UC San Diego, Altman Clinical and Translational Research Institute, 9500 Gilman Dr, La Jolla, San Diego, CA 92093 USA

## Abstract

**Background:**

The incorporation of implementation science approaches into tobacco control program delivery remains limited. Programs developed using these approaches, including co-creation, are often more feasible and sustainable, as they are designed to consider the operational and cultural context of an organization. This study utilized multiple implementation science approaches to co-create a tobacco cessation workflow within a Federally Qualified Health Center (FQHC), where tobacco use is disproportionately higher than the national average.

**Methods:**

In this study we conducted six structured meetings across two advisory groups between July-September 2025. Multiple implementation science methods (i.e., functions and forms , brainwriting premortem, and Practical, Robust Implementation and Sustainability Model (PRISM) assessments) were integrated to optimize the tobacco cessation workflow for future piloting, along with co-creation and evaluation methods (i.e., ethnographic observations, engagement survey) to evaluate advisory board engagement. Qualitative and quantitative data were collected and analyzed simultaneously using rapid qualitative analysis and descriptive statistics allowing for a comprehensive understanding and iterative incorporation of the results.

**Results:**

Thirteen individuals participated in this study as co-creation partners who were between the ages 27 to 69 years (M = 38.6). Participants represent various roles within the FQHC including front-line staff, administrators, providers, and patients. Workflow Optimization: Participants identified 5 core functions and 16 forms in the refinement of the tobacco cessation workflow. Brainwriting Premortem resulted in 28 unique failures and 19 solutions within the workflow across PRISM contextual domains, of which 16 were integrated. The final version of the co-created workflow had high acceptability (M = 4.8), appropriateness (M = 4.6), and feasibility (M = 4.5). Participant Engagement: A total of 394 interactions were identified across meetings. The most frequent interaction was giving information (53%), followed by seeking information (18%). The quality and frequency of advisory board engagement was rated favorably (average 4.7 of 5). Rapid qualitative analysis of open-ended responses highlighted the value of co-creation with participants stating: “I am glad I participated in this advisory panel… and I am able to bring [this information] back to my team to help our patients.”

**Conclusion:**

This study highlights how co-creation through multiple implementation science methods can be used to conceptualize, adapt, and refine programs responsive to FQHC needs. As formative, pre-implementation work, these findings reflect acceptability, feasibility and appropriateness of co-creation processes; real-world adoption and effectiveness remain untested and represent important next steps for future research.

**Supplementary Information:**

The online version contains supplementary material available at https://doi.org/10.1186/s43058-026-01038-2.

## Contributions to the literature

• As the use of implementation science frameworks in tobacco cessation remains limited, our study contributes to the literature by demonstrating how PRISM, alongside implementation science methods and co-creation, can rapidly refine clinical workflows to strengthen perceived feasibility, acceptability, and appropriateness.

• We provide one of the few systematic approaches to evaluating partner engagement and show how diverse partners contribute to distinct insights.

• Our study offers a design process that is transferable across diverse health service organizations and disease topics or conditions along with practical recommendations for researchers and practitioners interested in using co-creation methods in their work.

## Introduction

Despite persistent tobacco control efforts, the prevalence of smoking remains high in the United States, with significantly greater rates of smoking among low-income populations [1, 2]. These disparities are particularly evident among patients within Federally Qualified Health Center’s (FQHCs). FQHCs are non-profit community health centers typically located in medically underserved communities that offer primary care and preventative health services to communities disproportionately affected by economic, social, and healthcare inequities [3–5]. As of 2026, FQHCs provide care to approximately 52 million patients, 51% of whom were low-income or uninsured [6]. The prevalence of smoking among FQHC patients is 5% higher than the national average [5] and low-income populations predominately served by FQHCs are often less likely to be advised to quit tobacco use or be referred for tobacco treatment highlighting the potential high impact FQHCs can have on underrepresented populations when promoting tobacco cessation [5].

While effective interventions, such as the 5 A’s for tobacco cessation [7, 8] are well established, their integration into clinical care remains challenging. The literature has cited various barriers to integrating tobacco cessation into routine care including lack of time to discuss or prioritize tobacco cessation [9–11], limited knowledge/awareness of tobacco cessation guidelines [9, 11] lack of internal and external resources for tobacco cessation referrals [9], and limited organizational support for tobacco cessation [12]. These challenges are further amplified in FQHCs, which manage complex patient needs under finite resources. For example, providers may have limited time to address tobacco use when focusing on patients’ primary medical concerns, and limited awareness of available referral options can lead to missed opportunities for counseling or follow-up [13]. Importantly, because FQHCs vary widely in structure, staffing, and workflows, strategies that work in one setting may not translate directly to another. More research is needed to identify approaches that address these multilevel barriers while aligning with the unique contexts of FQHCs [14]. Thus, there is a need for strategies that take into account and align with individual, clinical, and organizational perspectives, preferences, and priorities to increase the likelihood of successful adoption and sustained implementation of evidence-based practices, such as tobacco cessation.

One promising approach to creating sustainable public health initiatives is through partner engagement. Meaningful partner engagement has consistently been recognized as an essential tool for public health programming [15–17]. Rooted in community engagement and user-centered design is “co-creation”, which brings together diverse partners to contribute to the planning, implementation, adaptation, and evaluation of an intervention [18, 19]. While outcomes research on co-created programs are newly emerging, there is evidence to suggest that co-created programs are designed to be more acceptable, appropriate and relevant, thereby increasing overall effectiveness and improving health promoting behaviors as they are designed to consider the operational and cultural context of an organization [20–22].

At SYHealth, the second largest FQHC in San Diego County, partner engagement and co-creation has played a pivotal role in advancing public health initiatives such as COVID-19 testing [23, 24] and vaccinations [25]. However, similar approaches have not yet been applied to tobacco cessation efforts. While SYHealth has high rates of tobacco screening (98%), tobacco cessation counseling falls to 68%, which is below the national average for community health centers at 72% [26]. Even more scarce is tobacco treatment, with only 23% of patients having a recorded tobacco treatment plan. These gaps underscore a pressing need to enhance tobacco cessation efforts at a large, multi-site FQHC, that can better align to individual, clinical and organizational contexts to support seamless integration.

Successful adoption and long-term implementation of public health interventions in real-world contexts, such as FQHCs, often require systematic and active approaches, combining various implementation strategies alongside meaningful partner engagement [19, 27]. To this end, this study applied multiple innovative implementation science approaches grounded in partner engagement and user-centered design [28, 29] to co-create a tobacco cessation workflow and implementation toolkit while also evaluating partner engagement. Advisory panels, a form of partner engagement, have frequently been used to engage diverse partners to inform research projects. These panels often provide feedback on the feasibility and acceptability of protocols, make recommendations for outcomes and milestones, and provide a “sounding board” for research ideas [30]. With the use of implementation science frameworks in the context of tobacco cessation being limited [31], integration of implementation science methods within the co-creation process, through advisory boards, is a promising approach. This integration can enhance the effectiveness, adoption, and long-term sustainment of tobacco control interventions. Furthermore, given the paucity of systematic evaluations of the quality and degree of engagement within advisory groups [32], our study employed an implementation science grounded evaluation of advisory board engagement, further advancing the literature. Thus, the objective of our study is twofold, (1) to describe our co-creation process of a tobacco cessation workflow and implementation toolkit guided by the Practical, Robust Implementation and Sustainability Model (PRISM) implementation science framework and (2) to report on methods and findings in evaluating partner engagement.

## Methods

### Study design

The study used convergent parallel mixed methods in which qualitative and quantitative data were collected and analyzed simultaneously allowing for a comprehensive understanding of the results [33]. Multiple methods from implementation science (i.e., functions and forms [34], brainwriting premortem [35], and PRISM assessments [36]) along with co-creation and user-centered design (i.e., partner selection and engagement where participants are actively engaged and lead the co-design process [37]) were integrated to iteratively develop a tobacco cessation workflow and implementation toolkit designed for SYHealth while also evaluating partner engagement. While the specific design of the workflows and toolkit are unique to SYHealth, the methods described below in the design process can be transferable across health service organizations and topics. The San Diego State University Institutional Review Board deemed the study not subject to IRB review. The study was approved for implementation by the partnering FQHC’s Research Review Committee (RHP-031124-169).

### Participants and recruitment

We would like to note that participants in this study refer to those individuals participating in the advisory boards as co-creation partners; thus, terms participant and partner are used interchangeably. All participants were recruited using a combination of convenience and purposive sampling strategies either being recommended by health center staff or were known to the lead author as an individual engaged in tobacco related work. We aimed to recruit 8–12 participants based on guidance from the Dissemination, Implementation, Communication, and Engagement toolkit for establishing advisory groups [30]. To promote diversity of perspective and relevance to the study aims, participants were selected based on either reported tobacco use (e.g., current or former smokers) or professional experience with tobacco screening and cessation counseling (e.g., providers and clinical staff) [30]. Eligibility criteria for patient members included being: (i) an active SYHealth patient; (ii) age 18 years or older; (iii) current or former tobacco/ nicotine product user, (iv) ability to participate in meetings virtually and/or in person. Eligibility criteria for all SYHealth staff members included being: (i) an active SYHealth employee; (ii) having primary functions that are patient facing and/or involve clinical site management or any involvement in implementing tobacco cessation efforts.

Twenty participants were invited to join one of two advisory boards via email or Microsoft Teams chat function of which 13 agreed. Half (*n* = 6) of the members formed the Tobacco Cessation Advisory Board, whose goal was to directly inform the co-creation and optimization of a tobacco cessation workflow and implementation toolkit tailored to the unique needs of SYH’s patient and organizational context. Another half (*n* = 7) formed the SYHealth Clinical Partner Advisory Board, whose goal was to identify failure points and mitigation strategies within the workflow co-created by the Tobacco Cessation Advisory Board to further enhance its acceptability, feasibility and appropriateness. We elected to have two distinct advisory boards to ensure that the tobacco cessation workflow was developed through a diverse lens and subsequently evaluated with objectivity by clinical partners responsible for its implementation.

Communication included a short description of the study, purpose of advisory group meetings, explanation of the study’s alignment both to the health center’s clinical goals and lead author’s (LS) interests in tobacco research, as well as logistical information such as approximate meeting dates, time, and duration. One week prior to the first scheduled meeting for each group, a confirmation email and study information sheet were sent to the participants. A reminder email was also sent ahead of each subsequent meeting. The Tobacco Cessation Advisory Board participated in 4 meetings which were scheduled for two hours on Zoom in the late afternoon/early evening. The SYHealth Clinical Partner Advisory Board participated in 2 meetings which were held in person for 90 min in the late afternoon. All meetings occurred weekly, were facilitated by the lead author (LS), and were recorded with participants’ consent. A description of each meeting is presented in Table 1.

**Table 1:** Overview of study phases, meeting goals, and participants engaged

Study Phase	Meeting Number	Meeting Goals	Participants
Phase 1	Meeting 1	Introductions & Project Overview Tobacco Cessation Workflow Co-Creation	Tobacco Cessation Advisory Board
Phase 1	Meeting 2	Tobacco Cessation Workflow Co-Creation	Tobacco Cessation Advisory Board
Phase 1	Meeting 3	Tobacco Cessation Implementation Toolkit Co-Creation	Tobacco Cessation Advisory Board
Phase 2	Meeting 4	Identify Potential Failures and Mitigation Strategies of Tobacco Cessation Workflow	SYHealth Clinical Partner Advisory Board
Phase 2	Meeting 5	Identify Contextual Failures for Implementing Tobacco Cessation Workflow and Implementation Toolkit	SYHealth Clinical Partner Advisory Board
Phase 3	Meeting 6	Validation of Tobacco Cessation Workflow and Implementation Toolkit and Assessment of Acceptability, Appropriateness, and Feasibility	Tobacco Cessation Advisory Board

### Data collection and analysis

The analysis team was a multi-disciplinary group of junior and senior researchers with diverse ethnic backgrounds and varying experience in public health, health care, and implementation science including the first author (LS) and senior implementation scientist (BR). Data collection occurred at two levels. The primary focus was to collect data using different implementation science activities (e.g., functions and forms, brainwriting etc.) and were facilitated through co-creation (e.g., discussions and observations) to elicit advisory board members input to optimize the tobacco cessation workflow and implementation guide. Simultaneously data was collected to assess the quantity and quality of partner engagement within the co-creation process. We will first present the measures and analysis for optimizing the workflow and implementation guide followed by the measures and analysis to evaluate partner engagement.

### Positionality & reflexivity

The lead author (LS) is a long-standing employee of SYHealth with significant experience developing, implementing and evaluating research programs over the last decade. She not only has familiarity with barriers and facilitators to implementing patient care workflows in high demand settings, but also has a deep understanding of available resources across organizational departments that can enhance workflow implementation. This deep organizational knowledge helped inform the study focus, which aligned with organizational priorities to improve tobacco cessation efforts, along with shaping the research question, methodology, and interpretation of results. Additionally, reflexivity was used to mitigate power dynamics and potential bias in the research process. For instance, as a Sr. Program Manager within the organization, the lead author recognized that this level of seniority could lead participants to provide socially desirable answers. To mitigate this, the lead author emphasized anonymity of the data and conducted meetings primarily over Zoom, outside of working hours. Regarding the data analysis process, the lead author actively engaged in reflexive dialogue and peer-debriefing with BR and integrated various data verification checks to ensure that interpretations were grounded in the data rather than influenced by personal biases or preconceived assumptions.

#### Workflow optimization measures and analysis

##### Functions and forms

As described by Jolles and colleagues, “functions” are the purpose of the evidence based practice (EBP) (i.e., what does the EBP seek to facilitate) while “forms” are the specific strategies to carry out those core functions (i.e., what are the activities needed implement the EBP) [34]. While functions are standardized, forms are customizable and adapted to the local context. Using this model, the Tobacco Cessation Advisory Board identified and operationalized the core tobacco cessation functions that can be standardized across SYHealth clinics as well as the key forms that can be tailored to specific sites as needed. Following the advisory board meetings, transcripts were generated using Zoom’s cloud recording function and reviewed against audio recording for accuracy by the lead author (LS). Rapid qualitative methods, a form of qualitative inquiry using triangulation and iterative data analysis [38] were used to identify overarching ideas generated by participants.

##### Brainwriting premortem

Brainwriting is an innovative method for group idea generation that harnesses the strengths of both individual ideation and collaborative exchange by emphasizing the exchange of ideas through written communication [39]. The Premortem approach was derived from a 1989 study which found that prospective hindsight – the act of imagining an event has already occurred – enhanced the accuracy of identifying future outcomes by 30% [40] and has more recently been used in the business context to understand program risks prior to actual implementation [41]. Brainwriting Premortem is a novel technique that combines these two approaches building on the combination of individual expertise and group problem-solving to identify failure points of project implementation and solutions for those failures, ultimately increasing the chance of program success [35].

The brainwriting premortem interview guide was informed by the protocols developed by Gilmartin and colleagues [35] and applied in research by Rabin and colleagues [32] and refined for this study to include three main components: (1) general overview; (2) brainwriting premortem to identify key reasons why tobacco cessation efforts would fail; and (3) solutions for the identified failure points. As the research team presented the draft of the co-created tobacco cessation workflow participants were asked to imagine that workflow failed at their respective clinic site and to think about why it failed. Participants were given a paper copy of the workflow to write their thoughts on during the presentation and were allotted an additional 15 min to continue writing down potential failure points silently. Each participant was asked to share the failures they identified. Failures were written on a whiteboard and organized into overarching groups when possible. If a failure was identified more than once, the recurrence was noted using a checkmark next to that specific item. Once all failures were reported, participants went through each failure and brainstormed potential solutions and mitigation strategies that could be adopted into the workflow to address that failure. Rapid qualitative analysis was used to describe the general characteristics of the failures generated (e.g., total number of failures generated) as well as solutions identified (e.g., number of solutions) and were organized in alignment with PRISM contextual domains.

##### iPRISM assessment

Participants were asked to complete a brief assessment on the feasibility of implementing the tobacco cessation workflow using the iPRISM webtool. The iPRISM webtool is an interactive tool intended to guide users through the process of aligning an intervention to the context in which it will be implemented [36]. The webtool is organized into four sequential steps: (1) Set up; (2) Assessment of Context; (3) Review of Assessment Results; and (4) Identifying and Prioritization of Implementation Strategies and Action Planning. The research team completed the set up and facilitated a discussion to answer the assessment questions in step 2. In this step, there are a total of 22 questions (16 operationalized to Reach, Effectiveness, Adoption, Implementation Maintenance framework (RE-AIM) implementation outcomes and 6 operationalized to the PRISM context domains). RE-AIM operationalized questions have six response options on a Likert scale from 1 “not at all likely” to 6 “very likely;” and PRISM operationalized questions have 6 response options on a Likert scale ranging from 1 “not aligned” to 6 “very aligned.” All questions also include a “not applicable” option. Once all the questions were answered, aggregated results were autogenerated and were reviewed with participants to then discuss potential implementation strategies to utilize for any domains that were scored low. Implementation strategies were ranked in terms of feasibility and impact on a scale of 1 (low feasibility/impact) to 4 (high feasibility/impact). Quantitative results from the iPRISM assessment were obtained through the webtool generated results and described descriptively. Qualitative results from discussion transcripts were analyzed using rapid qualitative analysis to highlight overarching themes or ideas.

##### Acceptability, feasibility, appropriateness survey

To assess acceptability, appropriateness, and feasibility, we used the Acceptability of Intervention Measure (AIM), Intervention Appropriateness Measure (IAM), and Feasibility of Intervention Measure (FIM) scales developed by Weiner et al. [42]. The self-reported questionnaire includes a total of 12 items, with four-items specific to each domain, measuring the extent to which the participant believes an EBP is acceptable (i.e., perception that the EBP is agreeable, palatable or satisfactory), appropriate (i.e., perceived fit, relevance or compatibility of EBP), and feasible (i.e., extent to which the EBP can be successfully carried out) within their organizational context [42]. The AIM, IAM and FIM are measured via a 5-point Likert scale where 1 is completely disagree and 5 is completely agree. Higher scoring indicates greater acceptability, appropriateness, and feasibility with no established cutoff scores [42]. The Cronbach alphas for the 4-item sub-scales for structural validity were: 0.85 for acceptability, 0.91 for appropriateness, and 0.83 for feasibility indicating high validity [42]. The test-retest reliability Cronbach alphas for the scales from the survey were 0.83 for acceptability, 0.87 for appropriateness, and 0.88 for feasibility indicating high reliability [42]. Results from the acceptability, feasibility, and appropriateness surveys were analyzed using descriptive statistics displaying the mean scores for each domain and open-ended response were analyzed using rapid qualitative methods.

#### Partner engagement measures and analysis

##### Ethnographic observations

We used a multi-method approach to documenting and assessing the quality and extent of member engagement within the Tobacco Cessation Advisory Board including documentation of the meeting process, post-meeting engagement survey, and participant reflections. Ethnographic documentation forms were adapted from previous studies and included information on various aspects of member participation including attendance, time spent speaking, modality used (computer/phone), arrival and departure times, along with who was speaking (i.e., sender) and type of interactions which included: (1) Seeking Information – asking for information from an individual or group; (2) Giving Information – providing unsolicited facts, data or opinions or providing information as a response; (3) Agreement – agreeing with or endorsing others statements or summaries; (4) Disagreement – disagreeing with or sharing an opposing view; (5) Summation – summarizing points and making conclusions; and 5) Closing – closing remarks or reflections [32]. Participants were informed of the dual role of the data collection in which the data were used to iteratively improve the engagement process and to describe the processes used to measure and evaluate engagement.

Descriptive statistics were used to summarize the following data from the observation forms: time spent speaking (i.e., minutes), modality used (i.e., computer, phone, or both), arrival and departure time, sender and types of interactions (e.g., seeking information, giving information). For analysis of interaction types, individuals were categorized by the group they represented (e.g., provider, administration, front-line staff, patient) and counts were generated for the number of each type of interaction (e.g., giving information) the sender engaged in. Rapid qualitative methods were used to analyze reflections made by participants at the end of each meeting and organized into overarching themes.

##### Engagement survey

At the end of each Tobacco Cessation and SYH Clinical Partner Advisory Board meetings, participants were asked to complete a validated measure assessing partner engagement developed by Goodman and colleagues [43]. Participants were asked to rate 9 statements assessing the quality (how well) and quantity (how often) of engagement through two prompts: 1) “how *well* do the partners leading research do the following” and “how *often* do the partners leading the research do the following”. Five response options for items assessing quality ranged from “poor” to “excellent” and five response options for items assessing quantity ranged from “never” to “always”. Both response options also included “not applicable.” An optional open-ended comment field was available for the sharing of any observations, comments, or suggestions related to the most recent partner meeting. The first instance of this survey also included questions related to demographics (e.g., gender, age, race/ethnicity, education level and years of experience with community engaged research). Using a 5-round Delphi process, the stakeholder engagement measure had high content validity [35] and has been successfully implemented with community and scientific advisory boards of similar composition [32]. Results from the survey were downloaded directly from Qualtrics and were analyzed after each meeting. Data were summarized using descriptive statistics which included mean scores on quality and quantity of engagement.

##### Data integration

**Fig. 1:**
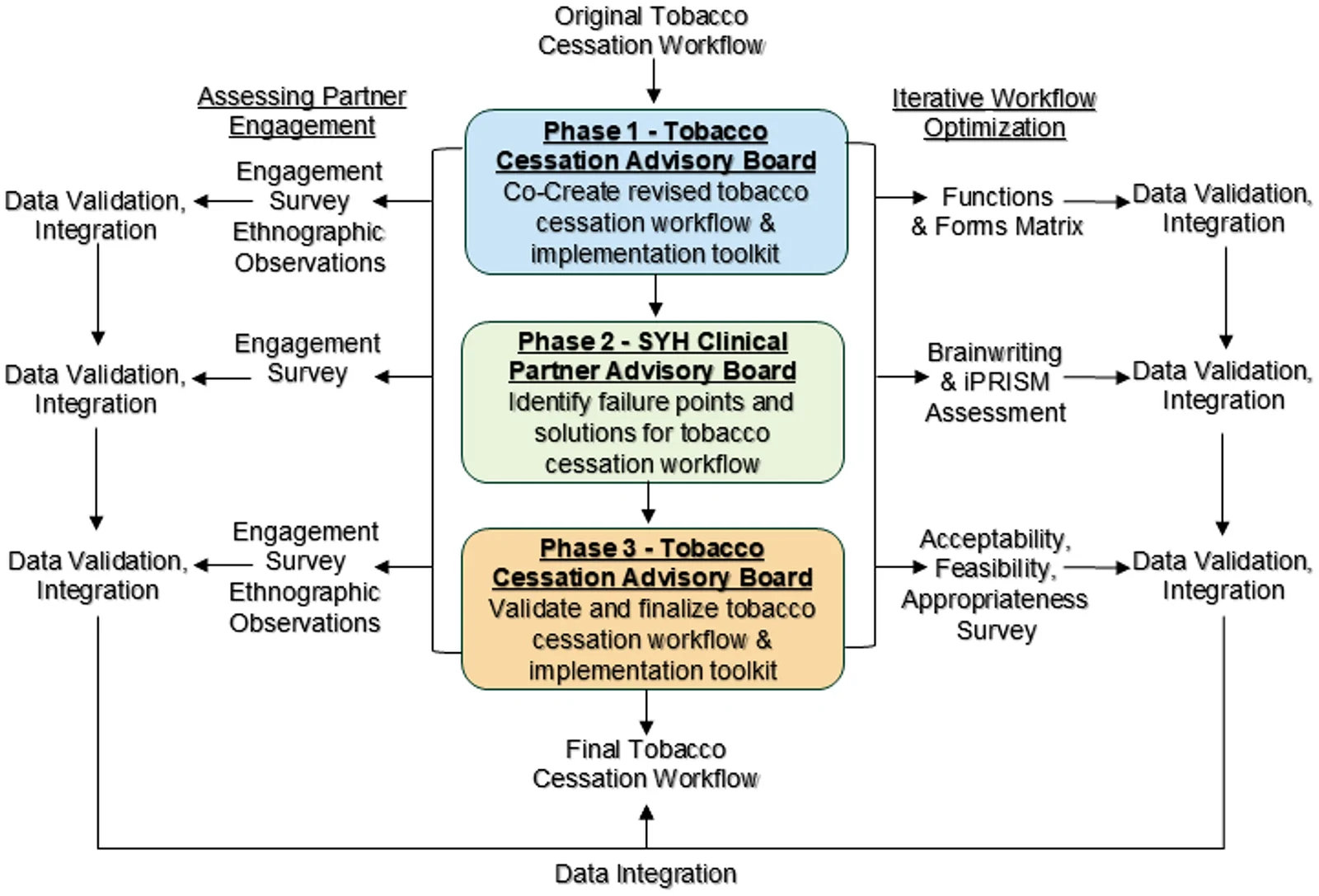
Data integration model

Data from multiple sources were integrated iteratively, and at multiple levels, throughout the study, as shown in Fig. 1, and were used to refine the tobacco cessation workflow and implementation toolkit. Importantly, given the dual role of the lead author as both facilitator and ethnographic observer, several iterative validation checks were integrated to ensure data was grounded in the participants experience.

The first level of integration occurred in Phase 1 through qualitative review of the functions and forms matrix. Meeting recordings were independently reviewed by the lead author and senior implementation scientist (BR). Key insights were synthesized into brief summaries by the lead author and were validated through peer debriefing with BR. Briefly, peer debriefing is a collaborative process involving sharing and discussing the data to support validation of interpretation [44] which encourages reflexivity [45] where researchers can reflect on their own biases and how their relationship with the participants, organizational, and data may influence their research. Upon confirmation of data interpretation refinements were made to the workflow and toolkit. The revised materials were presented to the advisory group at the following meeting for further validation prior to final adoption.

The second level of data integration occurred in Phase 2. Using the same validation approach, meeting recordings were independently reviewed by the lead author and BR. Qualitative results from the brainwriting premortem were summarized by the lead author, validated through peer debriefing, integrated into the workflow and toolkit, and reviewed by the advisory board for approval prior to final adoption. This refined version of the workflow then informed the iPRISM assessment which included both quantitative and qualitative measures. Qualitative results from the assessment were used to contextualize qualitative findings, resulting in a third layer of data integration.

The final integration occurred in Phase 3 where aggregated results from ethnographic observations and engagement surveys were analyzed alongside the quantitative results of the acceptability, feasibility, and appropriateness survey. This allowed us to explore how levels of engagement (i.e., participation, time spent speaking) and quality and quantity of engagement via specific domains (i.e., focus is on problems important to the community, community engaged activities are continued until goals are met) may have influenced perceptions of the tobacco cessation workflow and toolkit’s overall acceptability, feasibility, and appropriateness.

## Results

### Participant characteristics

Participants represented various roles within the FQHC with 6 Providers, 2 Administrators, 1 Registered Nurse, 1 Medical Assistant/Patient Services Representative, 1 Health Educator, 1 Research Assistant, and 1 Patient. Participants were mostly female (*n* = 10) and Hispanic/Latino (*n* = 9) and were between the ages of 27–69 years old (M = 38.6). Within participant roles, the SYHealth Clinical Partner Advisory board primarily included clinical staff (5 Providers, 1 Registered Nurse, 1 Health Educator) who are often responsible for implementing the workflow whereas the Tobacco Cessation Advisory Board included a broader range of perspectives (2 Administrators, and 1 Provider, Research Assistant, Medical Assistant, and Patient each) to support workflow development. Additional participant demographic characteristics are summarized in Table 2.

**Table 2:** Participant demographic characteristics (*n* = 13)

Demographic Characteristic	*N* (%)
**Age (mean**,** range)**	38.6, 27–69
**Community-Engagement Research Experience**	
1–2 years	5 (38.4%)
3–5 years	4 (30.77%)
6–11 years	4 (30.77%)
**Gender**	
Female	10 (76.92%)
Male	3 (23.08%)
**Ethnicity**	
Hispanic/Latino	9 (69.23%)
Middle Eastern	1 (7.69%)
White	3 (23.08%)
**Highest Level of Education Completed**	
Community College / Technical School	1 (7.69%)
College or 4 Years of University	6 (46.15%)
Graduate Schools	6 (46.15%)
**Primary Role**	
Administrator (Manager, Director)	2 (15.38%)
Provider (MD/DO)	6 (46.15%)
Registered Nurse	1 (7.69%)
Medical Assistant	1 (7.69%)
Research Assistant	1 (7.69%)
Health Educator	1 (7.69%)
Patient	1 (7.69%)

#### Workflow optimization results

##### Functions and forms

**Fig. 2:**
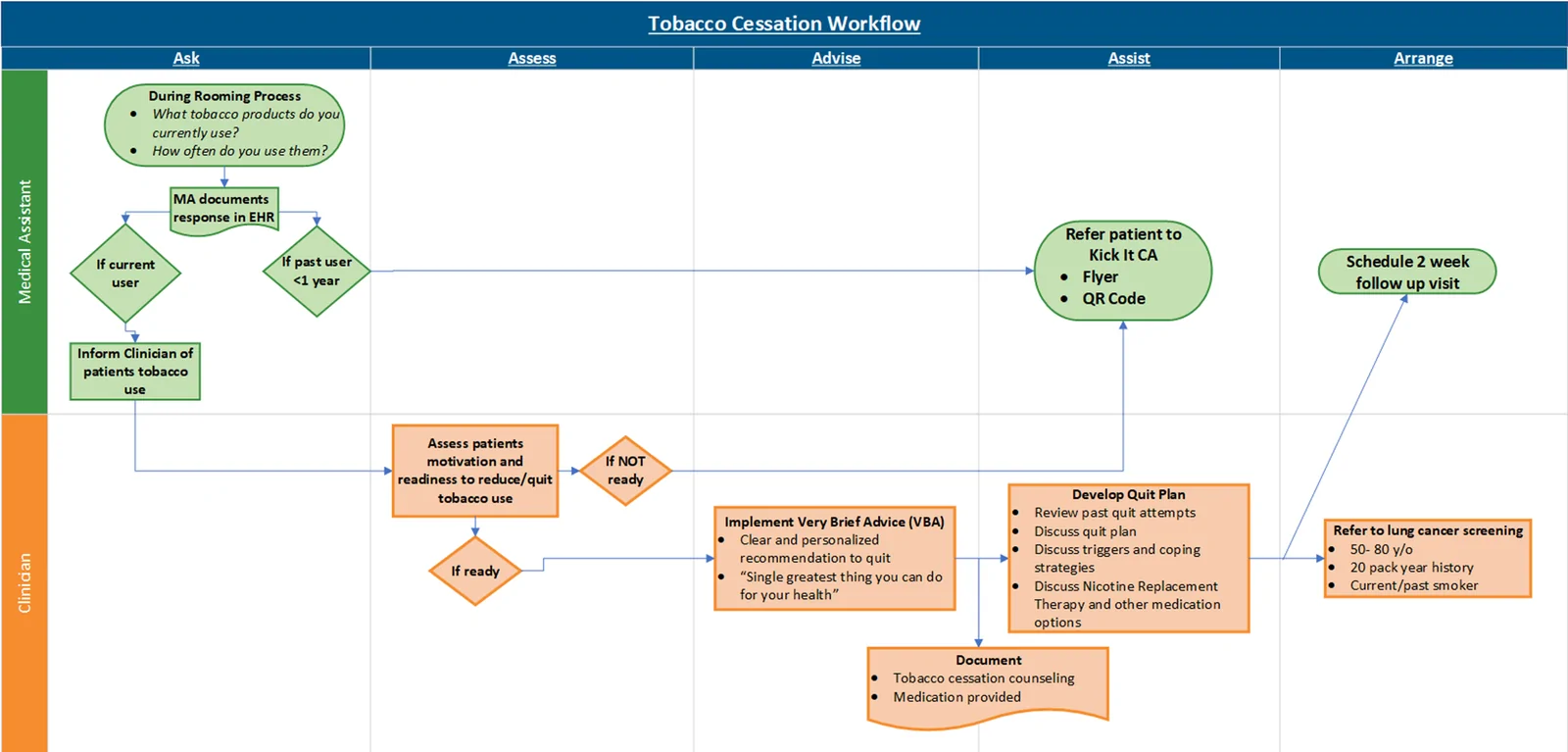
Tobacco cessation workflow – original

Participants in the Tobacco Cessation Advisory Board engaged in a series of three initial meetings to review the original tobacco cessation workflow (Fig. 2).

**Fig. 3:**
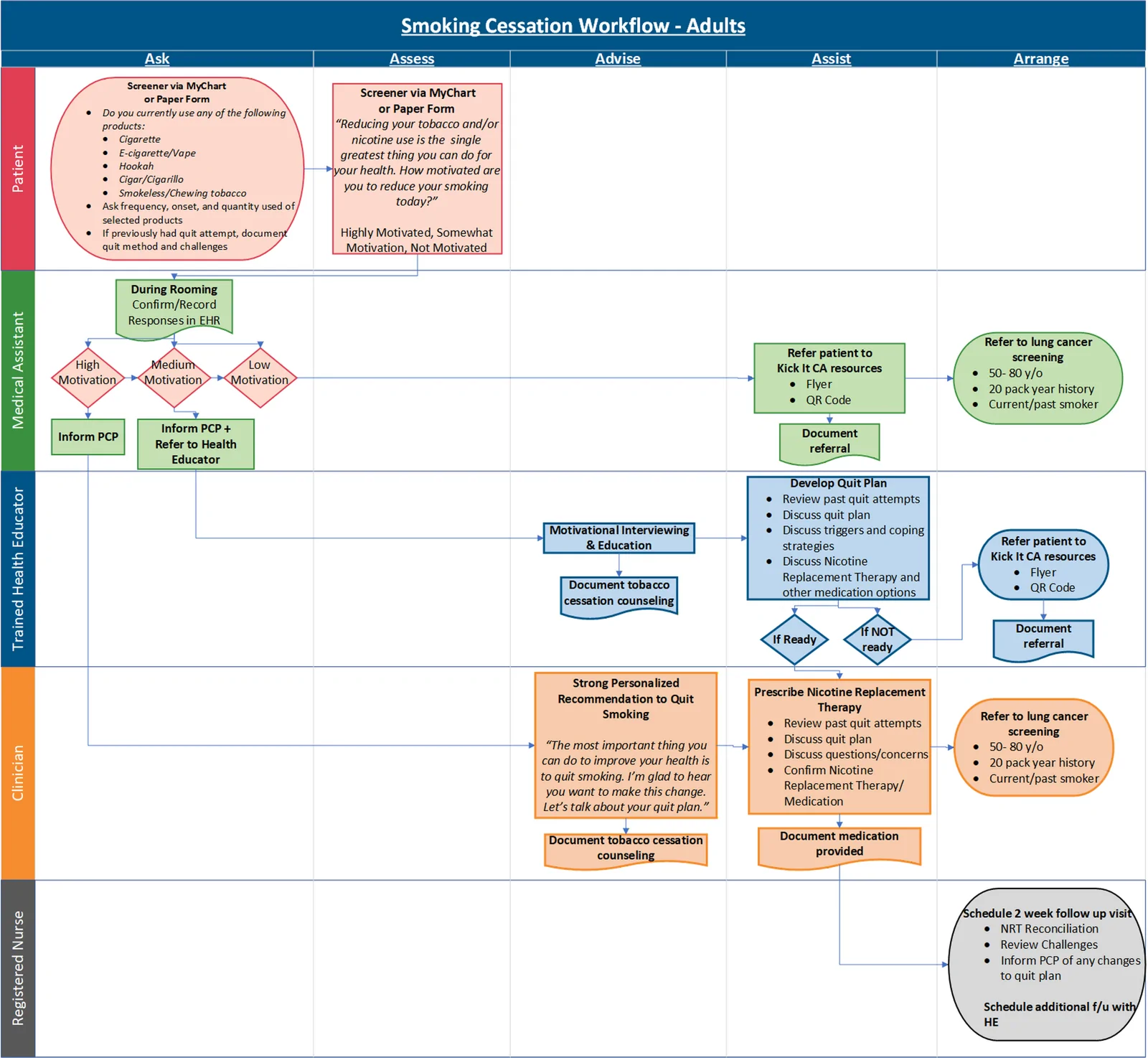
Revised tobacco cessation workflow - version 2

Participants confirmed the 5 core functions of the tobacco cessation workflow, following the 5’As model (Ask, Assess, Advise, Assist, Arrange). Within each core function, they discussed a motivating need or problem and identified a total of 16 new forms that could be tailored to SYHealth clinic sites and aligned to meet each core function. Table 3 displays these core functions, forms that could be adopted into the workflow, and who initially identified the specific form. Of the 16 new forms identified, the Tobacco Cessation Advisory group discussed and agreed to adopt 14 into the revised tobacco cessation workflow (Fig. 3). Forms that were not included in the workflow, along with additional recommendations (e.g., creating a pediatric specific workflow [Supplementary File 1]) were integrated into a tobacco cessation implementation toolkit, a detailed one-stop-shop document on how to integrate tobacco cessation efforts into a clinic along with recommendations for promoting tobacco cessation within and between departments (Supplemental File 2). The toolkit was reviewed twice with the advisory board (meeting #3 and #6) and participants were given an additional four weeks for independent review and feedback.

**Table 3:** Tobacco cessation workflow functions and forms matrix

Motivating Need/Problem	Core Function	Forms	Who Identified Form
Inconsistent Tobacco Use Screening	**Ask** every patient at every visit about their tobacco use	→ Patient responds to tobacco screening questionnaire prior to appointment	Administrator
		• Patient responds to tobacco screening questionnaire while in waiting room via tablet.	Administrator
		→ Patient responds to tobacco screening questionnaire while in waiting room via paper	Front Line Staff
		→ Medical Assistant completes tobacco screening questionnaire with patient while in exam room	Front Line Staff
		→ Include explicit questions around tobacco products (e.g., do you use vapes, do you use hookah?)	Patient
		• Screen patients for tobacco use during dental appointments	Front Line Staff Administrator
		• Medical Assistant screening during rooming process	(Existing)
Clarity needed on how to assess motivation to quit	**Assess** patients’ willingness to quit tobacco use	→ Patient responds to motivational assessment to quit tobacco during screening process	Patient
		Medical Assistant triages patient based on motivation to quit	Provider
		• Provider assesses motivation to quit tobacco use	(Existing)
Limited number of tobacco users advised to quit / Providers have limited time for counseling	**Advise** patients to quit using tobacco	→ Tobacco cessation counseling conducted by tobacco treatment specialist	Administrator
		→ Expand role of Adult Health Educators to support tobacco cessation counseling	Administrator
		• Provider initiates tobacco cessation counseling	(Existing)
Few tobacco users are referred to tobacco treatment	**Assist** patients in receiving behavioral counseling and/or pharmacotherapy to quit smoking	→ Refer to tobacco treatment specialist for behavioral counseling	Administrator
		→ Expand role of Adult Health Educators to support provision of behavioral counseling	Administrator
		• Expand roles of Registered Nurses to support provision of behavioral counseling	Provider
		→ Tobacco treatment specialist/Health Educator referral to local Quitline for behavioral counseling and pharmacotherapy	Patient
		• Medical Assistant referral to local Quitline for behavioral counseling and pharmacotherapy	(Existing)
		• Provider initiates behavioral counseling	(Existing)
		• Provider prescribes pharmacotherapy	(Existing)
Limited availability of provider appointments available to complete 2-week follow up	**Arrange** two-week follow-up with patients who initiate a quit plan and have a quit date	→ Schedule follow-up appointment with Registered Nurse	Provider Front Line Staff
		→ Schedule follow-up appointment with Tobacco Treatment Specialist	Administrator
		→ Schedule follow-up with Health Educator	Administrator
		• Schedule follow-up with Provider	(Existing)
		• Medical Assistant or Provider refers eligible patients to lung cancer screening	(Existing)

→ Indicates forms adopted into workflow

##### Brainwriting premortem

**Fig. 4:**
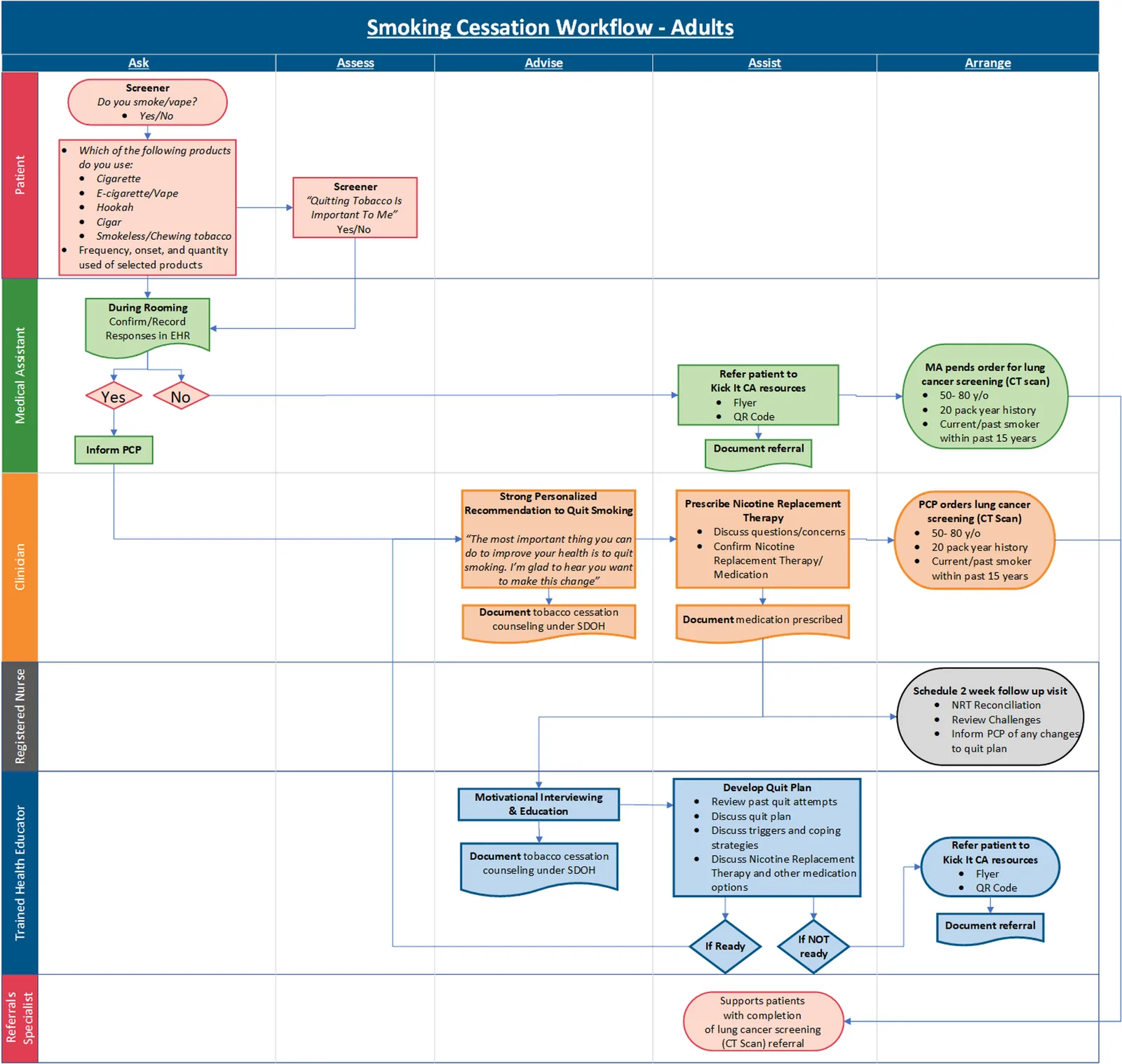
Revised tobacco cessation workflow - version 3

The SYHealth Clinical Partner Advisory Board engaged in a brainwriting premortem activity to identify potential failures of the revised tobacco cessation workflow. A total of 28 unique failures were identified along with 19 unique solutions to address and mitigate each of the failures aligning to each of the PRISM contextual domains. Table 4 highlights the unique failure, a description of the failure, solution proposed to address that failure, along with how the solutions were integrated into a new iteration of the tobacco cessation workflow (Fig. 4) or implementation toolkit.

**Table 4:** Overview of brainwriting premortem failures and solutions identified aligned to practical, robust implementation and sustainability model (PRISM) domains

PRISM Domain	Potential Failure	Failure Details	Solutions Proposed	How it was Integrated
Intervention (Organizational Perspective)	Communication breakdown between Primary Care Provider, Registered Nurse, and/or Medical Assistant	Lack of communication between staff supporting patients with tobacco cessation	Rapport building with staff Creating tobacco cessation communication pool (messaging inbox)	Integrated into implementation toolkit under Staffing & Capacity considerations
	Organizational buy-in on program	Organization lack of interest in engaging in tobacco cessation	Establish tobacco cessation as a care gap - track and share provider/clinic screening benchmarks to build accountability and engagement	Not Implemented
Recipient (Organizational Characteristics)	Patient tobacco use disclosure	Patients do not disclose or minimize tobacco use	Not Discussed	Not Applicable
	Failing to recognize tobacco use	Patients do not recognize certain products as tobacco use (e.g., hookah)	Using pictures and brand names to support connection between patients’ tobacco use	Not Implemented
	Patient inability to differentiate motivation to quit	Patients would have difficulty differentiating their motivation level between highly, somewhat, or not motivated	Use binary phrasing: “Quitting tobacco is important to me – Yes/No” to remove ambiguity in motivational assessment	Integrated into “assess” section of tobacco cessation workflow and implementation toolkit
	Patient lack of time to complete referrals	Patient’s lack of time to complete referral to Health Education or lung cancer screening	Not Discussed	Not Applicable
	Patient unwilling to do referral (x2)*	Patient’s unwillingness to follow up with lung cancer screening referral	Not Discussed	Not Applicable
	Patients buy-in	Patient’s lack of interest in engaging in tobacco cessation	Education - using flyers/video to ensure patients understand negative effects on smoking on their health	Integrated into implementation toolkit under Outreach & Awareness considerations
	Burden of intake questions (x2)*	Too many intake questions for patients to answer during visit	Obtain initial yes/no to “are you a smoker” and collect contextual details via Health Educator or Medical Assistant later	Integrated into “ask” section of tobacco cessation workflow and implementation toolkit
Recipient (Patient Characteristics)	Patient technology literacy	Patient’s inability to use MyChart to complete tobacco pre-screening	Use paper format or Medical Assistant screening to support patients who are not active MyChart users	Integrated into “ask” section of tobacco cessation workflow and implementation toolkit
	Patient low literacy (x2)*	Patient’s inability to understand screening questions	Education - using simple language or pictures to improve understanding and engagement	Not Implemented
	Patient language limitations (x3)*	Screenings questions only being available in English and/or not using common terminology	Offer screening questions in multiple languages to enhance accessibility	Not Implemented
	Patient medication non-adherence	Patient’s lack of medication adherence	Not Discussed	Not Applicable
	Recall bias on screening/ assessment questions	Patient’s inability to recall tobacco use onset, frequency, previous quit attempts	Not Discussed	Not Applicable
Implementation and Sustainability Infrastructure	Limited availability of follow-up appointments (x2)*	Limited availability of provider and nursing appointments to do tobacco cessation referral follow-up within 2-weeks	Primary Care Provider immediately discusses/prescribes nicotine replacement therapy and refers to Health Educator Establish tobacco cessation as a care gap - creates automatic referral to health education for further education	Integrated into “assist” section of tobacco cessation workflow and implementation toolkit Not Implemented
	Documentation of pack years (x4)*	Medical Assistant having challenges in accurately documenting patients pack year history	Education and training to ensure Medical Assistants properly document pack year history Establish tobacco cessation as a care gap - creates automatic prompts for routine screening and annual/biannual discussions - ensures discussion around tobacco cessation	Integrated into implementation toolkit under Staffing & Capacity considerations Not Implemented
	Lack of referral follow up	Limited availability of staff to follow up with patients for tobacco cessation, lung cancer, or other tobacco related referrals	Hire Referral Specialist to follow up with referral	Integrated into “arrange” section of tobacco cessation workflow and implementation toolkit
	Manual referral to health education	Provider time constraints to complete manual referral to health education	Establish tobacco cessation as a care gap - creates automatic referral to health education for further education	Not Implemented
	Treatment delay for “somewhat motivated” participants	Once patients are ready for tobacco treatment after Health Education, they still need to wait a long period of time to get follow up appointment with provider to obtain nicotine replacement therapy	Open prescription for nicotine patches for timely intervention	Integrated into “assist” section of implementation toolkit
	Patients not getting mailed referral	Referrals that are mailed do not reach patients due to incorrect information	Hire Referral Specialist to follow up with referral	Integrated into “arrange” section of tobacco cessation workflow and implementation toolkit
	Provider time constraints	Even with highly motivated patients, providers have limited time to discuss all aspects of tobacco cessation	Primary Care Provider immediately discusses/prescribes nicotine replacement therapy and refers to Health Educator Establish tobacco cessation care gap - creates automatic prompts for routine screening and annual/biannual discussions - ensures discussion around tobacco cessation -dedicate last 5 min of visit to discuss care gaps	Integrated into “assist” section of tobacco cessation workflow and implementation toolkit Not Implemented
	Translation of products	Product types not translated appropriately	Use common terminology when translating products	Not Implemented
	Unclear on process for lung cancer screening referral (x3)*	Unclear if provider is ordering lung cancer screening test or just providing referral	Medical Assistant can create pending order for lung cancer screening Establish tobacco cessation care gap - generate automatic alerts for provider to refer patient to lung cancer screening	Not Implemented
	Where does tobacco cessation counseling get documented (x3)*	Unclear where tobacco cessation counseling is documented (e.g., chart note, clickable button)	Make documentation easily accessible and clear	Integrated into tobacco cessation workflow and implementation toolkit throughout
External Environment	External resources with telehealth appointment	Unclear how to provide resources (e.g., flyers) with telehealth appointments	Create automatic After Visit Summary sent via text message or email with educational materials	Not Implemented
	Insurance barriers for nicotine replacement therapy (x4)*	Insurance not covering nicotine replacement therapies	Use open prescriptions for nicotine patches for timely intervention Education - ensuring Medical Assistants properly document pack year smoking history for insurance approval	Integrated into “assist” section of implementation toolkit Integrated into implementation toolkit under Staffing & Capacity considerations

*(x#) indicates the number of repeated instances failure was referenced

##### iPRISM assessment

**Fig. 5:**
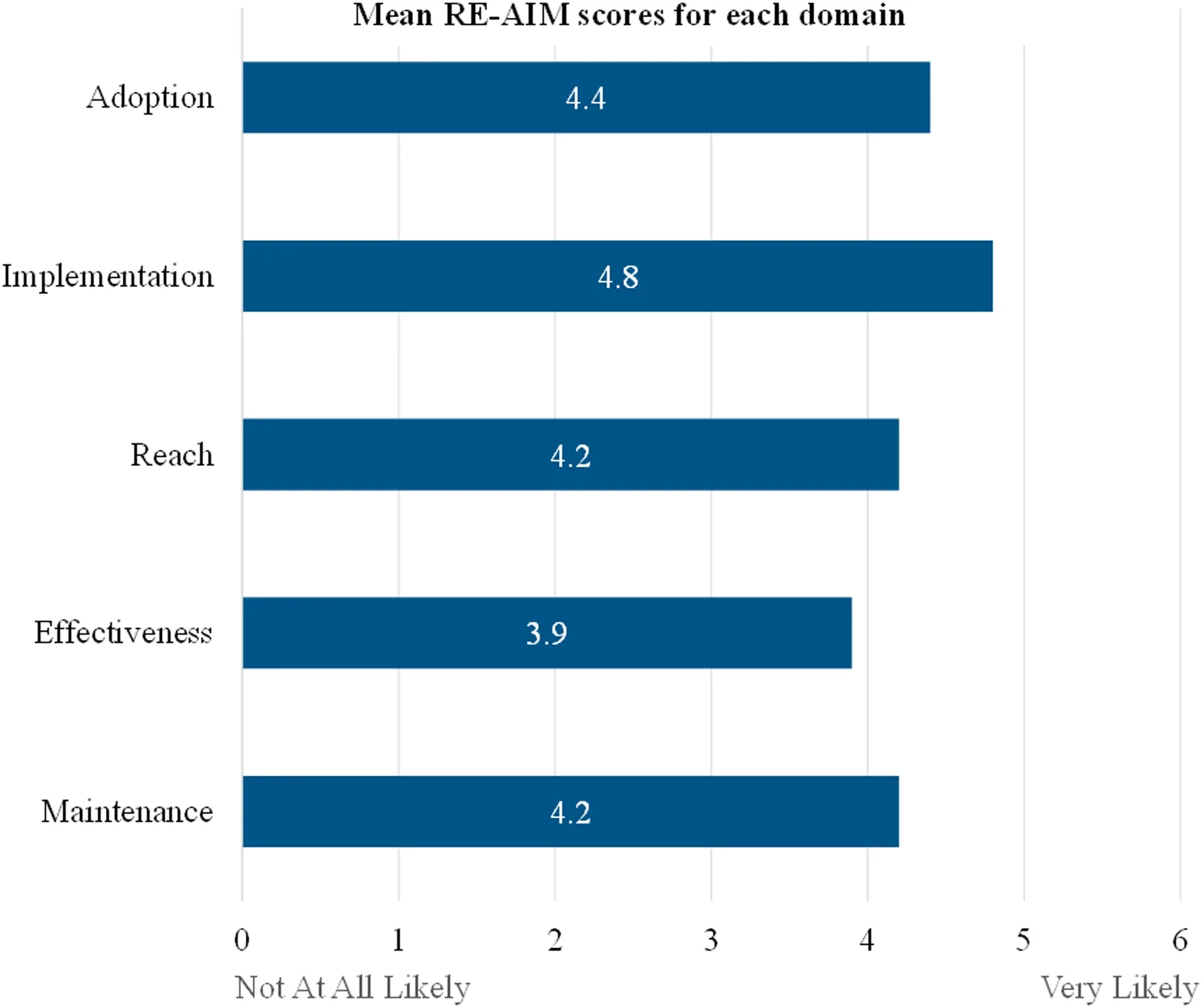
Mean RE-AIM scores for each domain (out of 6)

**Fig. 6:**
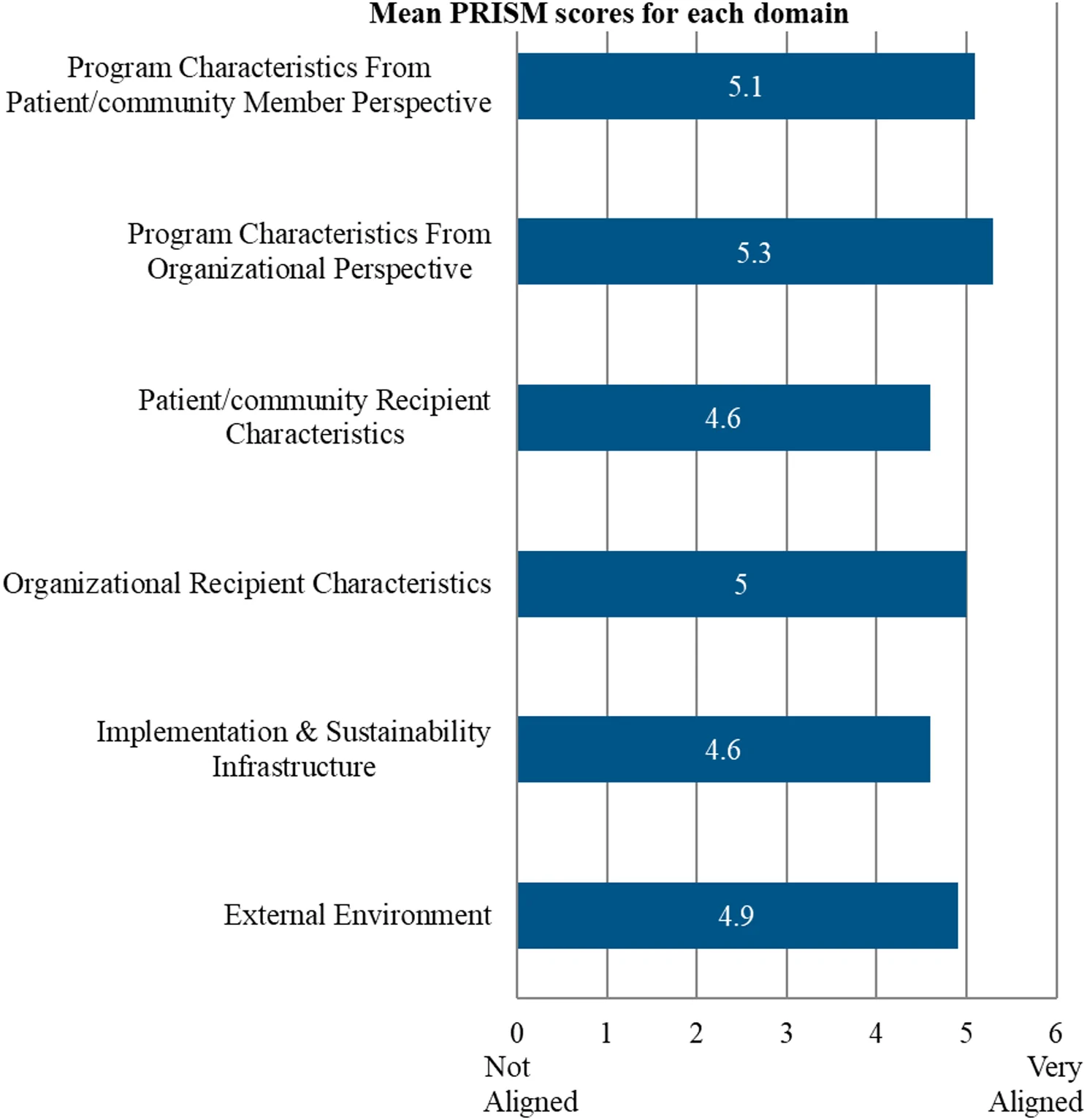
Mean PRISM scores for each domain (out of 6)

Prior to the iPRISM assessment, the SYHealth Clinical Partner Advisory Board reviewed and validated the adaptations in the 3rd iteration of the tobacco cessation workflow. Participants were then asked to think about the revised workflow in responding to the 22 questions aligning to the RE-AIM and PRISM constructs. Mean scores for RE-AIM measures ranged between 3.9 and 4.8 out of 6. Mean scores for PRISM measures ranged between 4.6 and 5.3 out of 6. Average scores from each domain are presented in Figs. 5 and 6. The two lowest scoring prompts included “the SYH Tobacco Cessation Program will be effective for intended recipients that are socially and economically disadvantaged?” within the effectiveness domain, scoring a 3.6 out of 6, and “the SYH Tobacco Cessation Program will continue to be delivered over time in a high percentage of participating settings?” within the maintenance domain, scoring a 3.9 out of 6.

Rapid qualitative analysis of discussion notes highlighted two themes “prioritization” and “consistency” as underlying reasons for the low scores. The SYHealth Clinical Partner Advisory Board collectively agreed that patients often have chronic co-morbid conditions that result in lack of tobacco cessation prioritization during clinical encounters. Additionally, challenges including transportation and financial constraints make it difficult for patients to prioritize seeking treatment for tobacco use. Thus, while the SYHealth Clinical Partner Advisory Board believed in the importance of tobacco cessation, they concluded that social and economic hardships would hinder potential effectiveness. Regarding maintenance, the SYHealth Clinical Partner Advisory Board highlighted that until tobacco cessation becomes an organizational care gap, the initiative would likely get lost as new priorities emerge. However, consistent training and visual reminders via care gap dashboards that indicate the percentage of patients who are screened, counseled, treated for a condition could mitigate this concern as tobacco cessation would remain at the forefront of clinical quality conversations.

Within these themes, the SYHealth Clinical Partner Advisory Board identified 5 implementation strategies through collaborative discussion that could support increasing the effectiveness and maintenance of the tobacco cessation workflow which included; (1) Patient education, (2) Hard stops in EHR for tobacco measures, (3) Ensuring over the counter nicotine replacement therapies are available in SYHealth pharmacies, (4) Establish tobacco cessation as an organizational care gap, (5) Ongoing tobacco cessation training and materials. The SYHealth Clinical Partner Advisory Board discussed these strategies in detail and, by consensus, ranked them based on their potential feasibility and impact to address identified challenges in the effectiveness and maintenance of the tobacco cessation workflow. Results from the rankings are presented in Table 5, with hard stops in EHR for tobacco measures ranked most feasible and highest impact. These strategies were then integrated into the tobacco cessation implementation toolkit as strategies that leaders can integrate to support overall tobacco cessation efforts.

**Table 5:** iPRISM implementation strategy feasibility and impact scores

Strategy	Feasibility score (out of 4)	Impact score (out of 4)
Patient education	3	2
Hard stops in EHR for tobacco measures	4	4
Ensuring over the counter nicotine replacement therapies are available in clinic pharmacies	4	3
Establish tobacco cessation as an organizational care gap	3	3
Ongoing tobacco cessation training and materials	3	2

##### Acceptability, feasibility, appropriateness survey

**Fig. 7:**
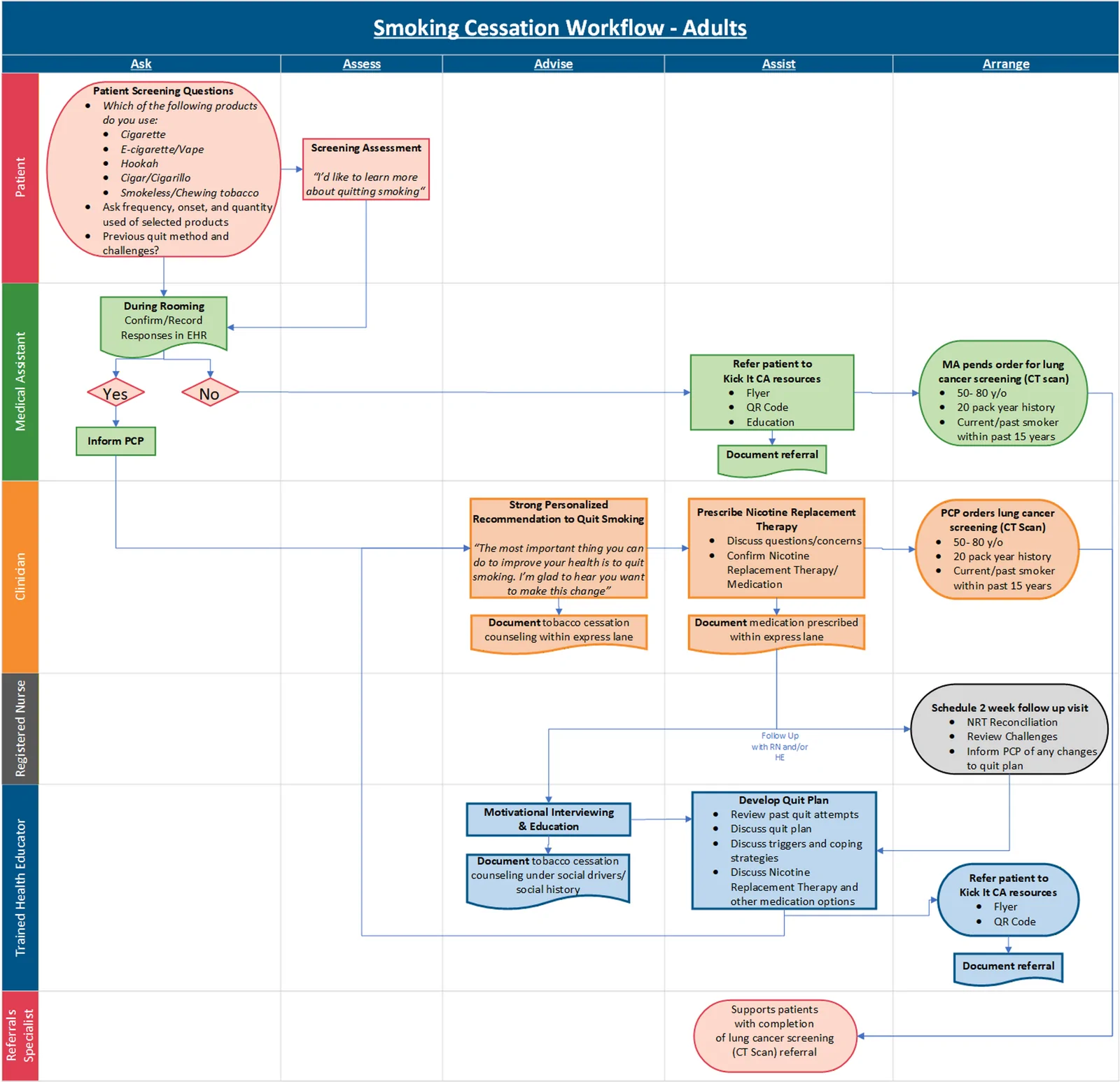
Final iteration of the tobacco cessation workflow – version 4

Version 3 of the tobacco cessation workflow was reviewed and validated by the Tobacco Cessation Advisory Board in the last meeting, in which final edits and clarifications were made. Version 4 (Fig. 7) was then assessed for acceptability, feasibility, and appropriateness. Results indicated high acceptability (M = 4.83) feasibility, (M = 4.50), and appropriateness (M = 4.66), of the revised tobacco cessation workflow. Results from the rapid qualitative analysis of open-ended responses identified three overarching themes that gave further context to the quantitative results: (1) Multiple represented perspectives; (2) Cautious optimism; (3) Use of multiple methods enhances appropriateness. Participants found the workflow to be acceptable given that it represented multiple perspectives. For instance, one participant stated, “The workflow is very well rounded-it shows that multiple stakeholders were carefully involved in the process.” Regarding feasibility, while ranked high, participants expressed cautious optimism, sharing that “variations in clinic capacities will ultimately determine how the workflow is adopted.” Lastly, participants shared that the use of multiple methods enhanced perceived appropriateness, with one participant highlighting that the “anticipation of barriers as well as the development of their potential solutions and workarounds, gives the workflow a solid foundation to become a sustainable implementation.”

#### Partner engagement results

##### Ethnographic observations

###### Overall engagement & time spent speaking

There was 100% participation among Tobacco Cessation Advisory Board members in all meetings. One participant consistently arrived late while another consistently left early due to conflicting schedules, though these were known in advance. All other participants joined for the duration of the 2-hour meeting. Among the Tobacco Cessation Advisory Board members, administrators spent the most time talking (85.25 min, 19.35% of total meeting time), followed by front line staff (81.18 min, 18.43% of total meeting time), provider (29.25 min, 6.64% of total meeting time) and patient (26.41 min, 5.99% of total meeting time). Speaking time generally increased across meetings. For instance, front line staff spoke for 9.44 min in the first meeting compared to 37.59 min in the last meeting. While smaller in scope, administrator speaking time also increased from 14.36 min in the first meeting to 25.30 min in the last meeting. Patient and provider speaking time was relatively consistent across meetings with an average of 6.40 min and 7.21 min, respectively, though patient speaking time did double (13.44 min) in the final meeting.

###### Interactions by group

Table 6 displays the different types of interactions documented across the four sessions. There was a total of 394 interactions documented across the Tobacco Cessation Advisory Board meetings. Giving information was the most frequent type of interaction followed by seeking information. Excluding the facilitator, administrators had the most instances of giving information, seeking information, and agreements followed by front line staff. Disagreements occurred infrequently and only among administrators and front-line staff and summations were only documented by the facilitator.

**Table 6:** Frequency of interaction type (in minutes) by tobacco cessation advisory board member type

	Seeking Info	Giving Info	Agreement	Disagreement	Summation	Closing Reflection	Total
Facilitator	54	71	3	0	20	7	155 (39.34%)
Administra-tors	9	61	24	4	0	7	105 (26.65%)
Front Line Staff	6	46	15	2	0	10	79 (20.05%)
Patient	3	15	11	0	0	5	34 (8.63%)
Provider	1	16	1	0	0	3	21 (5.33%)
Total	73 (18.53%)	209 (53.05%)	54 (13.71%)	6 (1.52%)	20 (5.08%)	32 (8.12%)	394

*Reflections*

Analysis of closing reflections identified three overarching themes: (1) Enhancing Self-Awareness to Integrate Tobacco Cessation; (2) Value of Co-Creation; and (3) Future Considerations that shed light to the experiences of participants within the co-creation process. Within the first theme, participants often highlighted that participation in the Tobacco Cessation Advisory Board meetings made them reflect on how they can better integrate tobacco cessation into their own work. For instance, one administrator stated, “[I’m] thinking about how in my role I can…include[e] tobacco cessation, as you know, a quality measure that we talk about, especially during our [quality] meeting” (Clinic Site Manager). Similarly, a provider reflected on the need to continue discussing tobacco cessation even after treatment has been provided to patients, stating: **“**I’m reflecting on how can I better support my current patients as I see them… not just like that first you know conversation, or when we talk about that, they’re motivated and ready, and we prescribe medication, but also like the follow up, because I feel like…once the patient has been prescribed medication, we don’t spend as much time with it anymore” (Provider).

Within the second theme, participants shared similar sentiments to the value that co-creation brings to program development. In particular, every participant reflected on the importance of having multiple perspectives in the workflow development process. A front-line staff member shared: “I learned a lot, which I enjoyed, especially hearing the perspectives from people that have different responsibilities in the organization and I think that that having those different perspectives was really evident in like the final product (Research Assistant). Relatedly, the process of co-creation was seen to enhance participants perceived feasibility of the tobacco cessation workflow with a participant sharing “‘I’m super excited about seeing all of it kind of come together, and it could actually [be] feasible, you know, that it can be implemented. So I’m very excited about all of this” (Patient). Lastly, participants shared various future recommendations such as integrating at least 1 in-person meeting, continuing to use Zoom platform for flexibility, considering alternative meetings times, and highlighting the importance of having a skilled facilitator which made the co-creation process engaging overall.

###### Engagement survey

Response rates for the engagement survey were high with 100% of participants completing the survey after each meeting. The quality and frequency of engagement was rated overall favorably (M = 4.82). Almost all participants (92%) reported good or excellent engagement across quality domains and often or always across quantity domains. Open-ended responses echoed themes identified in the reflection analysis, with participants noting that their involvement enhanced self-awareness and motivation to integrate tobacco cessation into their own work. One participant shared, “I am glad I participated in this advisory panel, and I am able to bring it back to my team to help our patients” (Registered Nurse). Others emphasized the value of co-creation, stating, “I think everyone is very committed to making sure we can improve/enhance patient care in this respect” (Medical Assistant). Taken together, these findings suggest that the advisory panel fostered meaningful engagement, reinforced participants’ commitment to tobacco cessation, and highlighted the value of collaborative approaches to improving patient care.

## Discussion

This study utilized multiple implementation science approaches alongside partner engagement and user-centered design to co-create a tobacco cessation workflow within a FQHC, where tobacco use is disproportionately higher than the national average. We successfully convened 6 advisory board meetings, bringing together a diverse group of clinical, administrative, and patient partners to develop, refine, and optimize the workflow for future piloting, while also systematically evaluating partner engagement. This co-creation process emphasized the use of action-driven techniques, which led to a rapid and efficient method of integrating feedback from multiple partners in a short timeframe, resulting in a positively rated tobacco cessation workflow and highly rated level of partner engagement. These formative, pre-implementation results represent the acceptability, feasibility and appropriateness of the co-creation process; however, future research is still needed to test the real-world adoption and effectiveness of the workflows and toolkit.

The focus on iterative and timely refinement of the tobacco cessation workflow through use of multiple implementation science techniques is a key component of this study. The field of implementation science has long recognized the importance of ensuring evidence-based practices have impact in real-world settings [46], alongside the need to accelerate the uptake of interventions into clinical settings [47, 48] Rapid assessment and feedback is essential to achieving these goals [49]. Building upon the emerging literature in iterative implementation science methods [49, 50] we employ similar methods through the lens of PRISM, using data to iteratively refine our tobacco cessation workflow, optimizing it for future piloting.

Each of our implementation science techniques (i.e., functions and forms, brainwriting premortem, iPRISM) allowed partners to identify unique challenges and opportunities to improve our tobacco cessation workflow. The rapid integration of feedback from our Tobacco Cessation Advisory Board on functions and forms served the foundation of the tobacco cessation workflow. The rapid assessment of brainwriting premortem results with our SYHealth Clinical Partner Advisory Board resulted in 28 unique failures to the refined workflow along with 19 proposed solutions across PRISM domains. The iPRISM webtool, which has shown to be an effective tool for assessing the fit of an intervention in hospital settings [51], was a novel technique used in this study to evaluate our tobacco cessation workflow which highlighted two low-scoring areas of effectiveness and maintenance of the workflow and identified 5 additional implementation strategies that could increase these outcomes. Refinement, validation, and integration occurred at the beginning of each meeting resulting in iterative and rapid optimization of the tobacco cessation workflow. Ultimately, the final version of the tobacco cessation workflow was found to have high acceptability (M = 4.83), feasibility, (M = 4.50), and appropriateness (M = 4.66) highlighting the importance and value of multi-method, multi-perspective, and iterative approaches when developing clinical processes.

Regarding partner engagement, engagement ratings across meetings were overwhelmingly positive, with 92% of participants rating the quality and quantity of engagement as good or excellent across all domains. Attendance was consistently high, with 100% of participants attending all meetings, and majority of participants arriving on time and staying for the entire duration of the meeting. When looking at time spent speaking, the total contribution between all Tobacco Cessation Advisory Board members was 222.9 min (50.4% of the total meeting times) accounting for half of the total time spent speaking. We expected that the researchers’ time spent speaking would account for a large portion of total speaking time as they would facilitate discussions, present results and processes, and summarize key points. These results are consistent with findings reported by Rabin et al., where while the facilitators accounted for a large portion of speaking time, the average contribution across advisory board members was 58% [32].

Notably, we found a general increase in speaking time across participant groups. Front-line staff nearly tripled the time spent speaking from the first meeting to last while administrators and our patient representative doubled their speaking time. We would like to note that lower speaking times of the patient and provider (i.e., patient on average spoke for 6.40 min) did not necessarily indicate a low level of engagement as there was only one individual within each of these roles while other roles (i.e., administrator) had multiple individuals. We also found that while “giving information” was the primary interaction type, participants were eager to “seek information” from their peers. A notable contribution was our addition of “disagreements” to the ethnographic observation documentation adapted from earlier work [32]. While only a few instances of disagreements occurred, we found it important that they were shared not only by administrators, but also front-line staff. In triangulating our engagement survey results with these interaction types, we believe that the high ratings for quality and quantity of engagement, specifically within the following domains: (i) All partners ideas are treated with openness and respect; (ii) The partnership’s processes support trust among all partners, and (iii) Mutual respect exists among all partners, contributed to an environment where members felt comfortable sharing their opinions, even if not in alignment with administrative leaders.

Qualitative analysis of reflections, open-ended responses from the engagement survey, and open-ended response from the acceptability, feasibility, and appropriateness questionnaire converged, further contextualizing quantitative results. Participants consistently highlighted the value co-creation brings, particularly the inclusion of multiple perspectives and multiple methods, to developing clinical programs. They also shared sentiments about how participation in this study enhanced their self-awareness and motivation to integrate tobacco cessation into their own work to support patient care. While some participants expressed caution, indicating that the level of adoption would be contingent on clinic capacities, the anticipation of barriers and identification of various solutions and implementation strategies provided a strong foundation for a sustainable program. These perceptions underscore that the focus on tobacco cessation was in alignment to the needs of the health center and its patients and likely contributed to the overall positive evaluation of the tobacco cessation workflow.

Our study fills two important gaps in the literature; (i) using well-known and novel implementation science methods to advance tobacco cessation efforts and (ii) systematically documenting and evaluating partner engagement. Importantly, this study also responds to concerns raised in the broader literature that co‑creation is often implemented without systematic evaluation, limiting what can be learned from these processes [52]. We use a multi‑method and iterative evaluation approach to understand how partner engagement enhances the co‑creation process. This approach mirrors recommendations by Peters et al., who emphasize that rigorous co‑design requires explicit attention to evaluating both internal (i.e. group and research processes) as well as considering broader contextual factors (i.e., systems, sustainability, and health outcomes) [52]. Attending to these multiple levels reinforces that co‑creation benefits most when diverse perspectives are integrated to capture the full range of factors shaping implementation. Within our study we found that across advisory boards, different partner groups contributed distinct forms of expertise that filled important knowledge gaps in the planned implementation of the tobacco cessation workflow. For example, the SYHealth Clinical Partner Advisory Board partners identified 28 previously unrecognized failures and 19 solutions during the brainwriting premortem, which were not raised by the Tobacco Cessation Advisory Board. The patient partner highlighted the importance of patients being able to self‑assess their motivation to quit tobacco use during the core function of “assess,” which was not recognized by other partners. These examples illustrate that each group surfaced different considerations that were essential for refining the workflow, emphasizing that co‑creation within a single role (e.g., only medical providers, or only patients) cannot anticipate all barriers and facilitators needed to ensure that interventions are meaningful, and more likely to be feasible and well‑received.

Despite the benefits of the co-creation process, we do note that our study does have some limitations. Firstly, while we recruited a diverse group of members, we did not have representation from pharmacists or dentists. As research on the effectiveness of improving tobacco cessation through the use of pharmacies and dental visits is growing [53, 54] understanding unique challenges and opportunities to optimize tobacco cessation processes within these settings warrants further research. Secondly, we recognize that every FQHC is unique and thus our developed products may not be generalizable to other settings. However, the methods used can be transferable to a wide range of disease topics and conditions important to health centers. We also acknowledge the potential of social desirability bias. It’s possible that because advisory board members were patients and employees of the health center, they rated the workflow in a more favorable light. Our team encouraged transparency and honesty and given the members openness to discuss disagreements and identify multiple failures, we expect that this bias had a small effect on our results. Given the lead authors dual role as facilitator and ethnographic observer, we recognize the potential validity concern as the facilitator is shaping the interactions being documented. To mitigate this, several safeguards were put into place to ensure that the data and adaptations of the workflows were grounded in the advisory boards’ experience rather than the facilitators’ perspective. A senior researcher who was not involved in facilitation independently reviewed session recordings and validated the observational findings of the lead author. Additionally, all findings and proposed adaptations were presented to the advisory board for member validation prior to final adoption. This iterative validation process ensured that interpretations accurately reflected participants’ perspectives. While independent observers are ideal when feasible, our use of multiple strategies for data validation substantially can reduce bias for researchers taking on dual roles. Additional limitations of our study include the use of open-ended survey responses, which may have constrained our ability to fully contextualize quantitative findings. We encourage future research to integrate brief qualitative interviews to better understand the experiences of partners in the co-creation process. We were limited to one patient perspective within the Tobacco Cessation Advisory board. While we believe we captured their perspective in the workflows given their strong engagement and increased participation overtime, we recognize that one patient may not represent the various knowledge, attitudes, behaviors, or values of all health center patients. Thus, having additional patient representatives could have balanced the overall advisory board composition. Finally, the co-creation process required substantial time and resources, which may pose challenges for replication in resource-constrained settings. Despite these limitations, our study underscores the value of co-creation and implementation science to advance tobacco cessation efforts in FQHCs.

The next stage of this research will be to conduct a small pilot to demonstrate preliminary effectiveness of the adapted workflows and toolkits. Given SYHealth’s robust Research and Health Promotion department, there is capacity to conduct a small unfunded pilot. Organizational leadership has been supportive of the pre-implementation workflow development and has prioritized improving tobacco cessation efforts across the organization. Pre-pilot efforts will focus on identifying potential electronic health record changes, understanding and optimizing access to nicotine replacement therapies, along with automated clinical dashboards for reinforcing maintenance would be prioritized. Additional considerations prior to pilot implementation would be to review the five implementation strategies identified by advisory board members and determine which are most feasible to deploy during the pilot phase to directly address low perceived effectiveness and maintenance. For example, obtaining over-the-counter nicotine replacement therapy for distribution within SYHealth pharmacies or integrating tobacco screening, cessation, and referral quality metrics into clinic dashboards may be two high-impact, feasible strategies that could strengthen these outcomes. Subsequently, the pilot phase will include collaboratively identifying 2–3 pilot sites with strong clinic level buy-in matched with comparable control sites based on size, patient population, and baseline tobacco use and cessation rates. Guided by the RE-AIM framework, the pilot will assess Reach (e.g., number, proportion and representativeness of SYHealth patients in the intervention), Effectiveness (e.g., changes in screening, cessation counseling, nicotine replacement therapy referrals/prescription, and changes in smoking patterns), Adoption (e.g., number, proportion, and representativeness of clinicians implanting the intervention), Implementation (e.g., degree to which workflow is delivered as intended). Additional data collection will include patient satisfaction surveys, cognitive interviews with staff to identify unforeseen challenges and opportunities for further optimization and a modified iPRISM assessment to evaluate whether perceived barriers related to effectiveness and maintenance have shifted after workflow implementation. These steps will allow us to further refine the workflow and assess early implementation feasibility prior to broader scale-up and organizational adoption and maintenance of the workflow across all sites. Beyond informing our own next steps, this work also offers several practical recommendations for researchers and practitioners interested in using co-creation methods in their work.

1. **Employ multiple methods to engage partners** to surface challenges and generate context-specific solutions that optimize implementation outcomes.
2. **Seek out diverse partner representation** to incorporate multiple perspectives.
3. **Assess engagement iteratively throughout the co-creation process** to identify low-scoring domains early and adapt facilitation strategies in real time.
4. **Use rapid methods** to integrate feedback and real time and support iterative product refinement.
5. **Capitalize on momentum** by scheduling weekly meetings, when possible, while ideas are fresh and motivation is high.

## Supplementary Information

Below is the link to the electronic supplementary material.

Supplementary Material 1

Supplementary Material 2

## Data Availability

The datasets generated and/or analyzed during the current study are not publicly available due organizational policies, but may be available from the corresponding author on reasonable request.

## Abbreviations

EBP: Evidence Based Practice
AIM: Acceptability of Intervention Measure
IAM: Intervention Appropriateness Measure
FQHC: Federally Qualified Health Center
FIM: Feasibility of Intervention Measure
FQHC: Federally Qualified Health Center
IRB: Institutional Review Board
PRISM: Practical, Robust Implementation and Sustainability Model
SYH: San Ysidro Health
