# Supplementary material for: Multi-method approaches to develop and refine a tobacco cessation workflow within a federally qualified health center
Source: Implement Sci Commun. 2026 Jul 6;7:205. doi: 10.1186/s43058-026-01038-2 (PMC13688938; doi:10.1186/s43058-026-01038-2)
Supplement: Supplementary file 1 [file 43058_2026_1038_MOESM2_ESM.pdf]

San Ysidro Health's Tobacco Cessation Implementation Toolkit

Note: This toolkit has been co-created by members of our Advisory Board to support tobacco cessation efforts. It represents work in progress and has not yet been formally adopted by the organization. We recommend health service providers or researchers to use this document as an example for developing similar toolkits of need.

Table of Contents

Overview: 5

Purpose of this Toolkit 5

The 5A's of Tobacco Cessation 6

Ask 7

Who and When to Screen 7

How to Screen: 7

Documentation Guidance & Suggested Language 7

Measures of Succes: 9

Assess 10

Who and When to Assess 10

How to Assess Motivation to Quit 10

Documentation Guidance 12

Measures of Succes: 13

Advice 15

Who to Provide Tobacco Cessation Counseling to 15

How to Advise Effectively 15

Documentation Guidance 16

Measures of Succes 17

Assist 19

Who to Assist in 19

How to Develop a Quit Plan 19

Documentation Guidance 22

Measures of Success 22

Arrange 23

Who to Arrange Follow-Ups For 23

How to Schedule Follow-Up Appointments 23

Documentation Guidance 23

Measures of Success 23

Site-Level Considerations for Clinic Managers 25

1. Quality Improvement & Data Review 25
2. Outreach & Awareness 26

3. Staffing & Capacity 27
4. Cross Departmental Integration 28
Tobacco Cessation Counselor Job Description - Example 30
References 33

Overview:

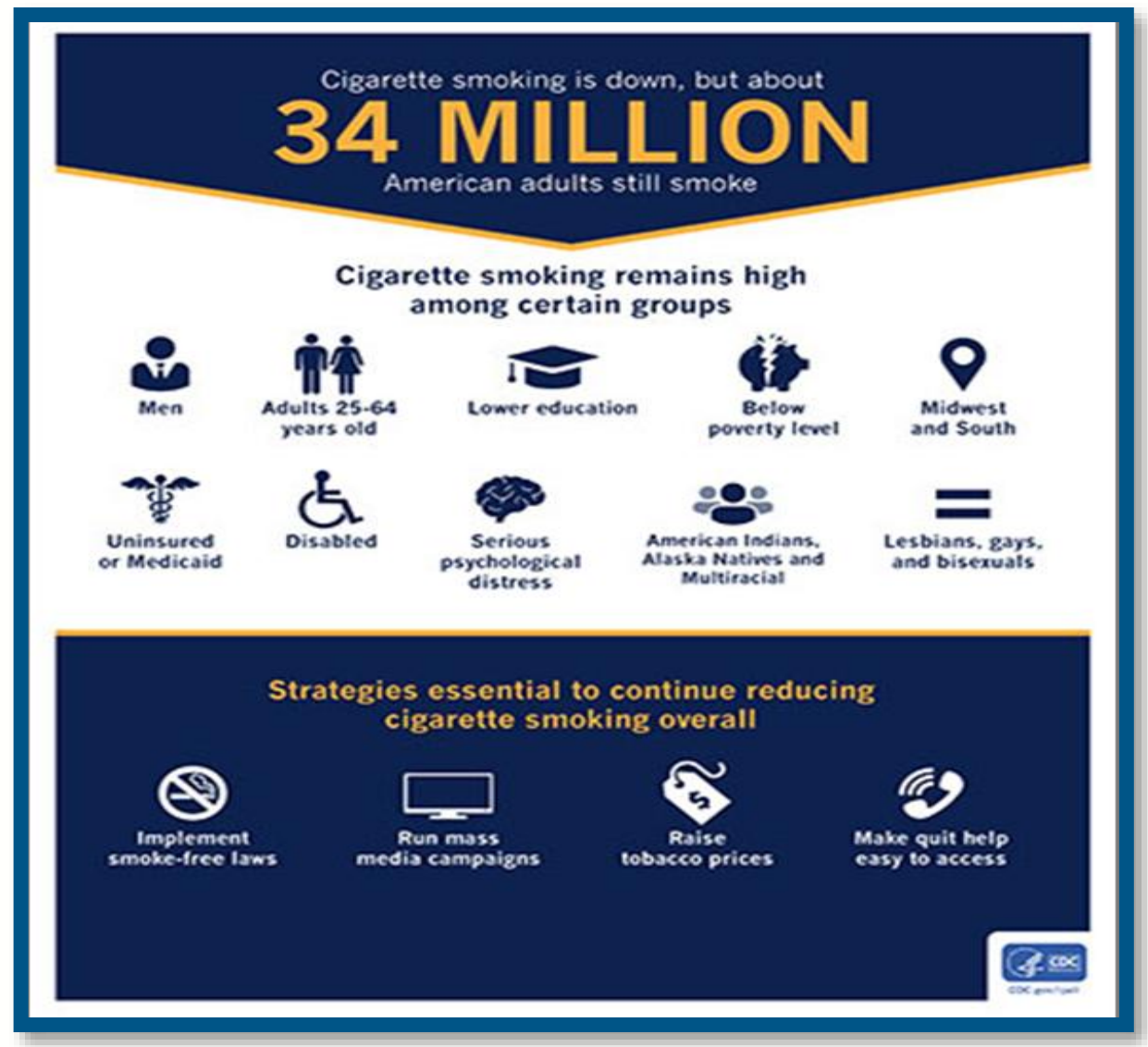

Tobacco is the leading cause of preventable death in: the United States.[1,2] Despite long-standing public health efforts, smoking rates remain disproportionately high in low-income communities.[2–4] Federally Qualified Health Centers, which predominantly serve these populations, report smoking prevalence rates more than **five times higher than the national average**, making them a critical setting for tobacco cessation intervention. [2,5,6]

Brief, provider-delivered advice is one of the most cost-effective and impactful cessation strategies.[7] However, it is often underutilized due to persistent barriers—such as limited time, gaps in training and awareness, resource constraints, and inconsistent organizational support—that prevent clinicians from routinely offering cessation counseling. [8–12]

San Ysidro Health Metrics: At SYHealth, tobacco screening is well-documented at **98%**. However, **cessation counseling drops to 68%**, falling below the national average for community health centers.[13] **Treatment initiation is notably lower**, with only **27%** of current smokers having a documented prescription for cessation support.

Purpose of this Toolkit: This guide provides SYH staff with a **pragmatic, team-based approach** to implementing a tobacco cessation program using the **5A's Framework**—Ask, Advise, Assess, Assist, and Arrange—endorsed by the U.S. Preventive Services Task Force.[14] While a single clinician can deliver all five components, dividing responsibilities among care teams is often more feasible and efficient.

Included in this toolkit:

- Key implementation steps (who, when, how)
- Documentation guidance
- Suggested phrasing for patient interactions
- Measures of success
- Site-level considerations for clinic managers

Together, these resources aim to make tobacco cessation a routine part of equitable care delivery across SYH settings.

The 5A's of Tobacco Cessation[7]

Ask about tobacco use
- Identify and document tobacco use status for every patient at every visit

Assess readiness to make a quit
- Is the tobacco user willing to make a quit attempt at this time?

Advise to quit
- In a clear, strong, and personalized manner, urge every tobacco user to quit.

Assist in quit attempts
- For the patient willing to make a quit attempt, offer medication and provide or refer for counseling or additional treatment to help the patient quit.
- For patients unwilling to quit at the time, provide interventions designed to increase future quit attempts.

Arrange follow-up
- For the patient willing to make a quit attempt, arrange for follow-up contacts, beginning within the first week after the quit date.
- For patients unwilling to make a quit attempt at the time, address tobacco dependence and willingness to quit at next clinic visit

Note: For **pediatric/adolescent participants** staff should continue to **follow the guidance for screening, assessing, and advising to reduce/quit tobacco use.** These patients should also **discuss a quit plan** that ***does not*** **include nicotine replacement therapy** and a **referral to Kick It CA** should be made for those ages 13 or older.

Reference: U.S. Department of Health and Human Services. *Smoking Cessation: A Report of the Surgeon General*.; 2020. https://www.ncbi.nlm.nih.gov/books/NBK555596/table/ch6.tab1/

Ask

Who and When to Screen:

Make tobacco use a vital sign. Ask **every patient** about tobacco use starting at age 11. Identify, verify, and document the tobacco use status of every patient at **every visit.**

How to Screen:

1. **For patients with active MyChart accounts**
   a. Participants should complete smoking screening questionnaire via MyChart in their pre-visit questionnaire.

2. **For patients without active MyChart accounts**
   a. Provide smoking screening questionnaire via tablet or paper while patient is in the waiting room.

3. **During Rooming Process**
   a. **Medical Assistants** should confirm responses to screening questionnaire are correct in MyChart and/or conduct the screening directly during the rooming process if screening has not yet been completed.

Documentation Guidance & Suggested Language:

Scenario	Medical Assistant Role
**Screening done before rooming**	**Verify and confirm** responses are accurately documented in the EHR.
**Screening during rooming**	**Ask and document** smoking history directly in the EHR.

1. Navigate to the **Social Drivers** within the EHR
2. Click on **Tobacco Use and E-Cigarette Vaping**.
3. Documenting patient history of using Tobacco Products.

   a. **Ask**
      i. "*Which of the following products do you use?*"
         1. Cigarettes
         2. Vapes/E-cigarettes
         3. Cigars/Cigarillos
         4. Hookah
         5. Chewing or smokeless tobacco

Proposed Enhancement

b. **Document** onset, frequency, and past quit attempts (for each product)

i. "When did you start using [selected product]"

ii. "How frequently do you use [selected product]"

1. If a cigarette user, enter approximate packs smoked per year

iii. "Have you ever tried to quit using [selected product]"

1. If yes, "when was the last time you quit using [selected product]"
2. "What medication and or treatments did you try when you last attempted to quit using [selected product]
    a. "Why do you think that [medication/treatment] was not successful"

c. **For pediatric/adolescent patients**

i. Ensure all documentation is completed with the confidential note

<u>SYH Current Screening Documentation</u>

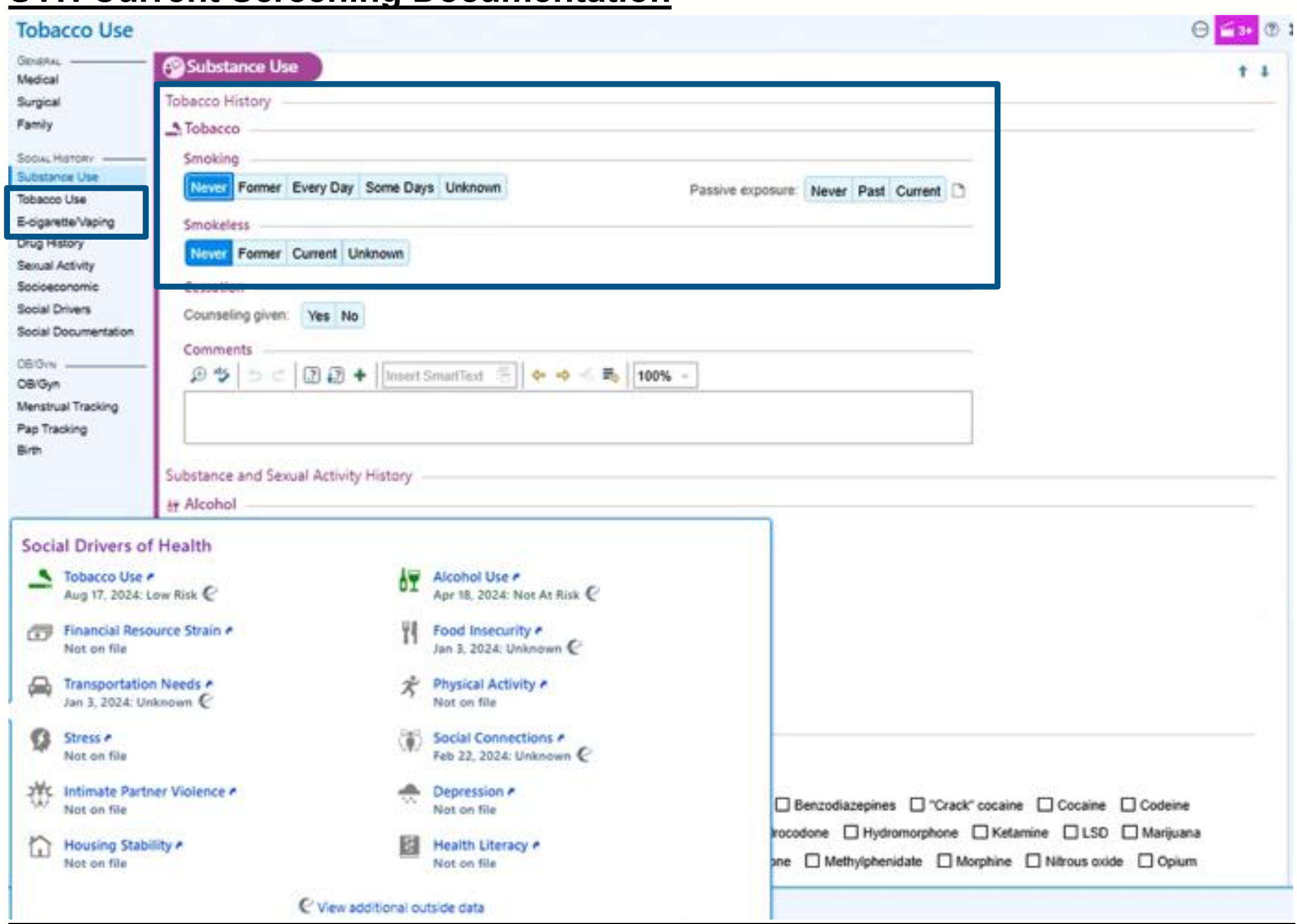

<u>Proposed Enhancements:</u>

1. Question: "Which of the following products do you use?"
    a. [list each product]

b. Patients should select or indicate all that apply

2. Types: Additional product types to be added include
    a. E-cigarette
    b. Cigarillos
    c. Hookah
    d. Smokeless/chewing tobacco

Measures of Succes:

1. Prior to implementation of screening workflow, Clinic Site Manager's should pull the Tobacco Cessation Report within EPIC dashboards to see their clinics baseline screening level.
    a. Reports can be stratified by participant socio-demographics to identify current trends.
    b. Baseline report should then be compared to new reports to assess changes in screening rates

Measure	Description	Target Benchmark
**Screening Completion Rate**	% of patients at each clinic site who have been screened for tobacco/nicotine use	≥ 95%
**Screening Equity**	Review by socio-demographic and organizational factors such as age, race/ethnicity, language, site, etc.	Identify gaps

Assess

Who and When to Assess Patients Motivation to Reduce/Quit Smoking:

All patients who use tobacco and nicotine products should complete a self-assessment of their current motivation to reduce or quit tobacco use. Motivation should be assessed during the screening process.

How to Assess Motivation to Quit:

1. Patients should respond to the following prompt: ***"I'd like to learn more about quitting smoking?"***
   a. Response Options:
      i. Yes
      ii. No

2. Responses should be documented in the EHR using one of the following pathways:
   a. **For patients with active MyChart accounts**
      i. Participants should complete motivation to quit assessment via MyChart in their pre-visit questionnaire.
   b. **For patients without active MyChart accounts**
      i. Provide motivation to quit assessment via tablet or paper while patient is in the waiting room.
   c. **During Rooming Process**
      i. **Medical Assistants** should confirm and/or document interest to learn about quitting smoking responses

Motivation Based Actions:

Motivation Level	Action	Follow-Up
**Yes**	Medical Assistant alerts PCP.<br><br>PCP reinforces quitting and prescribes appropriate treatment.	PCP documents discussion and treatment plan via tobacco cessation express lane.<br><br>PCP initiates referral for Health Education/RN for education and support.

**No***	PCP provides brief motivational message.	Leaves door open for future conversations and notes it in EHR.

***Refer to Advise section for additional resources**

Suggested Language based on Motivation Level:

Motivated	Speaker	Scripted Language
**Yes**	PCP	"The most important thing you can do to improve your health is to quit smoking. I'm glad to hear you want to make this change. Let's talk about treatment options that can help."
**No**	PCP/MA	"Quitting smoking is the most important thing you can do for your health. A referral to Health Education is a great place to start. When you're ready to begin treatment, I'll be here to support you."<br><br>"I understand that quitting smoking can be difficult. Here are some resources that can help you start thinking about it."

***Patients ages 13 and above**, regardless of motivation level, should receive Kick It California referral information.

Kick It California QR Code for SYH Referrals:

1. Patents can scan the QR code or copy the link to their browser to register to get free coaching from Kick It CA.

https://kic.ucsd.edu/Home/HLCI6291

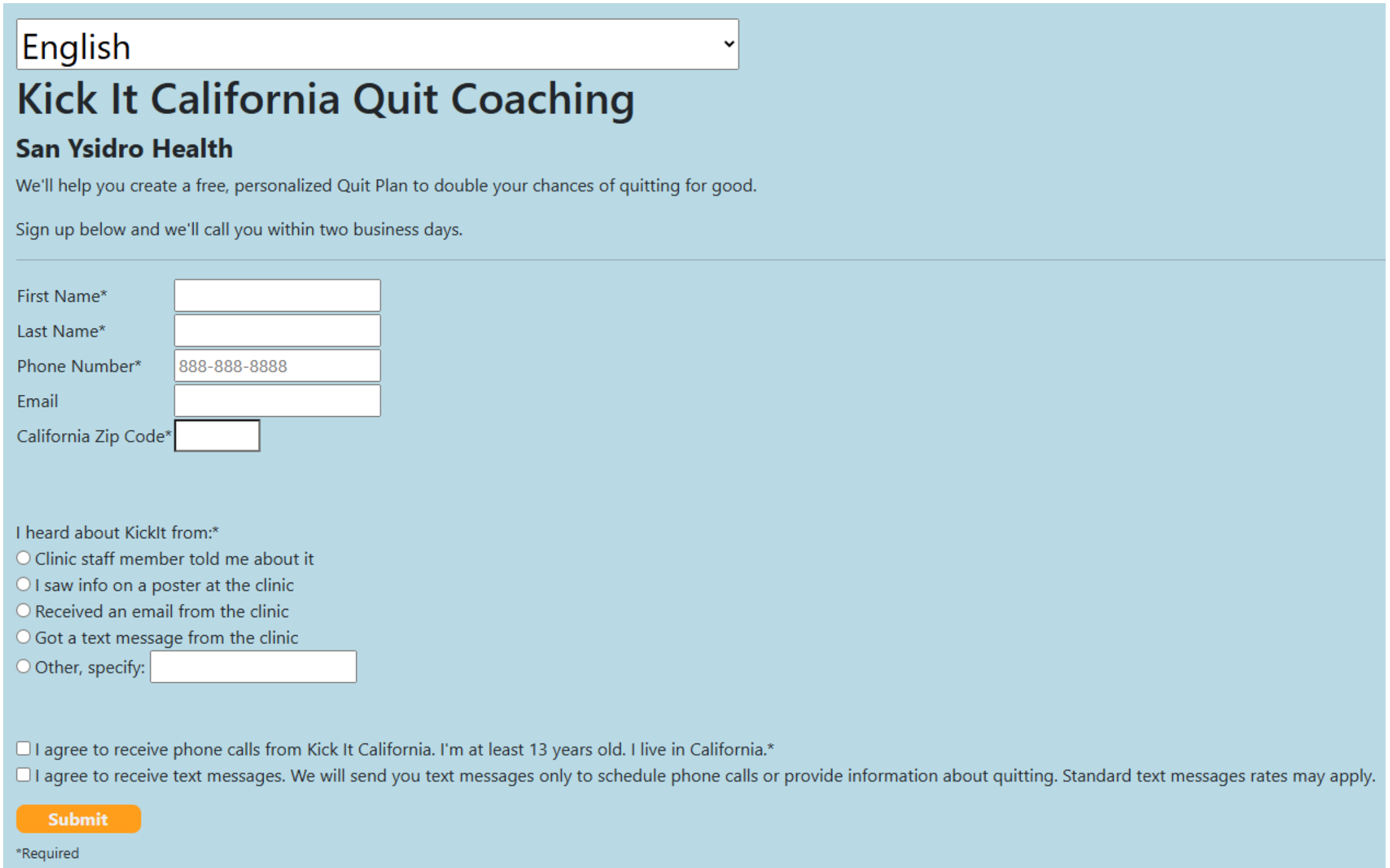

English

Kick It California Quit Coaching

San Ysidro Health

We'll help you create a free, personalized Quit Plan to double your chances of quitting for good.

Sign up below and we'll call you within two business days.

First Name*

Last Name*

Phone Number* 888-888-8888

Email

California Zip Code*

I heard about KickIt from:*

- Clinic staff member told me about it
- I saw info on a poster at the clinic
- Received an email from the clinic
- Got a text message from the clinic
- Other, specify:

- I agree to receive phone calls from Kick It California. I'm at least 13 years old. I live in California.*
- I agree to receive text messages. We will send you text messages only to schedule phone calls or provide information about quitting. Standard text messages rates may apply.

Submit

*Required

Documentation Guidance:

1. Navigate to the **Social Drivers** within the EHR
2. Click on **Tobacco Use and E-Cigarette Vaping**.
3. Document "ready to quit" Yes/No

AND

4. Navigate to the **Rooming tab** within the EHR
5. Scroll down **or click the Vital Signs**.
6. Document "ready to quit" Yes/No
    a. For **pediatric/adolescent patients**:
        - Ensure all documentation for is completed with the confidential note

SYH Current Assessment Documentation

1. Social History Documentation

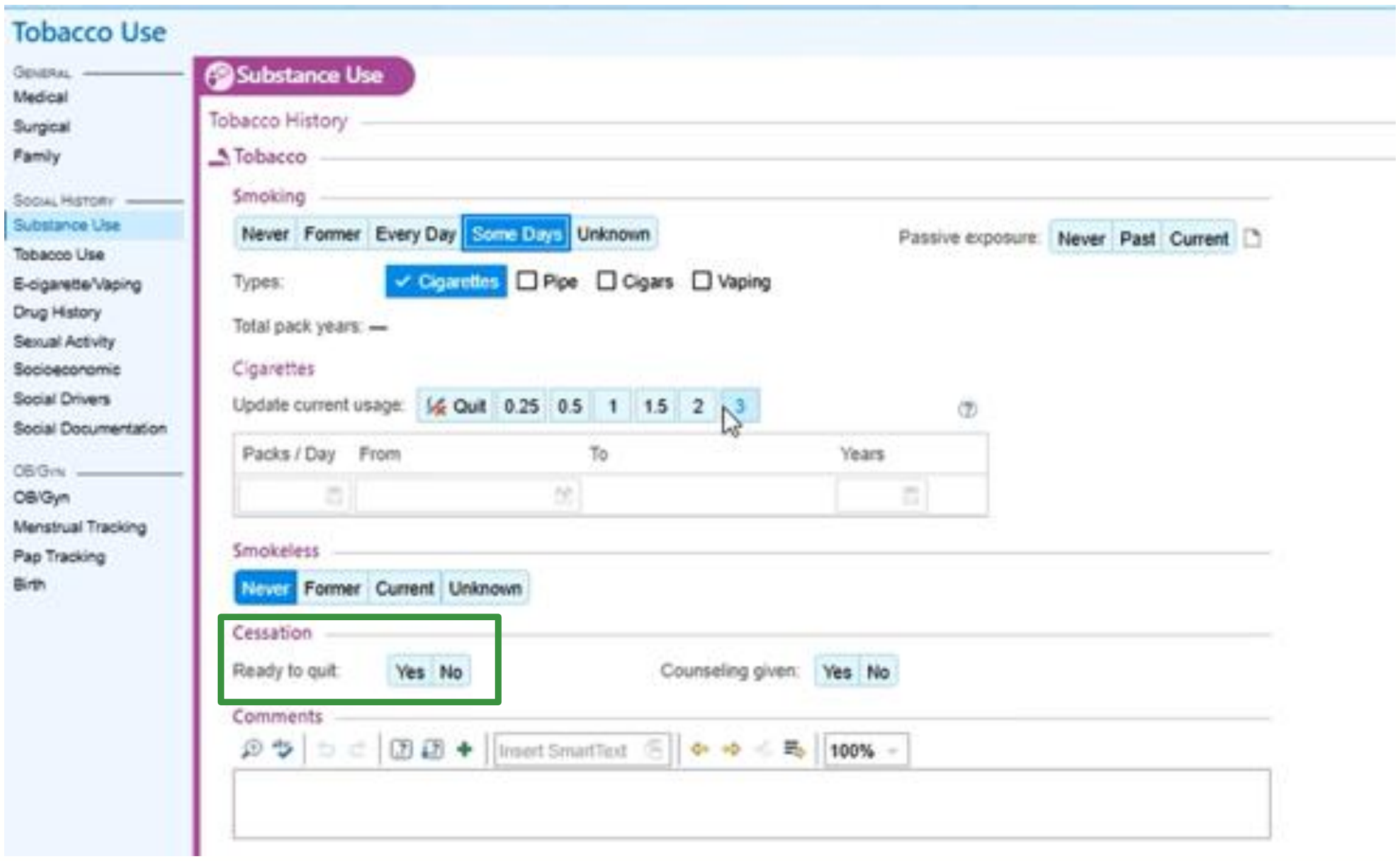

2. Rooming Tab Documentation

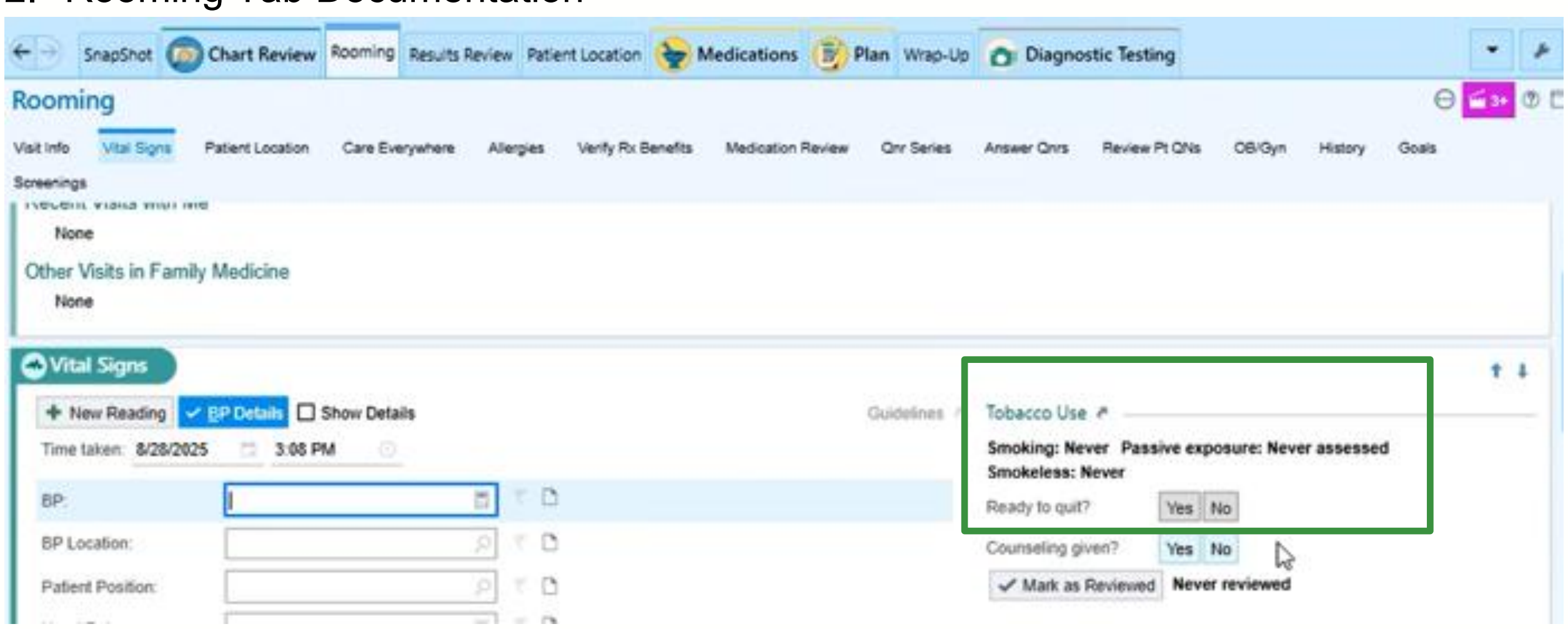

Proposed Enhancements:

1. Add motivation to quit assessment prompt "Quitting Tobacco Is Important to Me."

Measures of Succes:

1. Prior to implementation of assessment workflow, Clinic Site Manager's should pull the Tobacco Cessation Report within EPIC dashboards to see their patients baseline motivation to quit smoking levels.
   a. Reports can be stratified by participant socio-demographics to identify current trends.

b. Baseline report should then be compared to new reports to assess changes in motivational readiness

Measure	Description	Target Benchmark
**Motivational Readiness Documentation**	% of smokers with documented motivational readiness assessment response	≥ 95%
**Motivational Readiness Assessment**	% of smokers with their respective motivation level	Monitor for trends
**Kick It CA Referral Rate**	% of tobacco users given Kick It CA QR codes/flyers	≥ 95%
**Demographic Reach**	Review by socio-demographic and organizational factors such as age, race/ethnicity, language, site, etc.	Identify gaps

Advice

Who to Provide Tobacco Cessation Counseling to:

Tobacco cessation counseling should be provided every patient who uses tobacco and nicotine products. Advice must be **clear**, **strong**, and **personalized** to the patient's health, values, and context.

"The most important thing you can do to improve your health is to quit smoking—and I can help you."

How to Advise Effectively:

1. Effective tobacco cessation advice should be:
   a. **Clear:** *"It is important that you quit smoking/using tobacco now. Even occasional or light use is still harmful."*
   b. **Strong:** *"As your [role], I need you to know that quitting smoking is the most important step you can take to protect your health. Our team is here to help."*
   c. **Personalized:** Connect tobacco use to the patient's health status, chronic conditions, household exposure, or social/emotional impact*: "Continuing to smoke is worsening your asthma. Quitting could dramatically improve your symptoms."*

*For *patients with medium and low motivation* care team can consider the 5R model[14,15]

Component	Purpose	Examples / Prompts
**Relevance**	Encourage the patient to reflect on why quitting is personally meaningful.	Link to chronic illness, family health, pregnancy, prior quit attempts, or financial impact.
**Risks**	Help patients identify the health and social consequences of smoking.	Asthma, respiratory infections, heart disease, and secondhand smoke harms to family and children.
**Rewards**	Highlight the personal benefits of quitting tobacco.	Improved health, smell/taste, saving money, better role modeling for children, increased self-esteem.

**Roadblocks**	Identify and address perceived barriers to quitting.	Withdrawal symptoms, fear of failure, social triggers, lack of support, depression, weight gain.
**Repetition**	Reinforce that quitting often takes multiple attempts.	Revisit conversation regularly, praise continued interest, normalize setbacks as part of the process.

Documentation Guidance:

1. Navigate to the **Social Drivers** within the EHR
2. Click on **Tobacco Use and E-Cigarette Vaping**.
3. Document "counseling given" Yes/No

AND

4. Navigate to the **Rooming tab** within the EHR
5. Scroll down **or click the Vital Signs**.
6. Document "counseling given" Yes/No
    a. For **pediatric/adolescent patients**:
        - Ensure all documentation for is completed with the confidential note

SYH Current Counseling Documentation

1. Social History Documentation

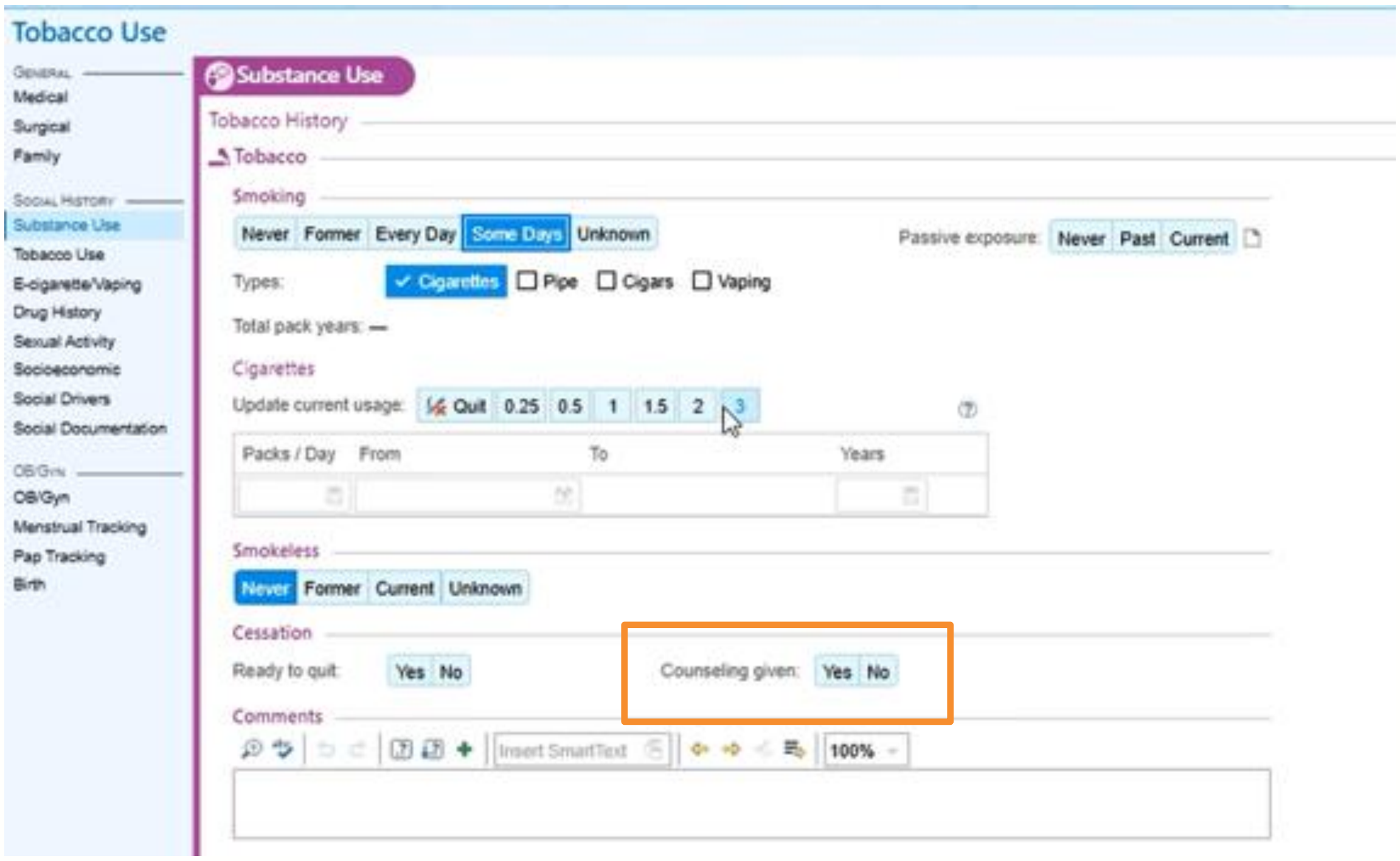

2. Rooming Tab Documentation

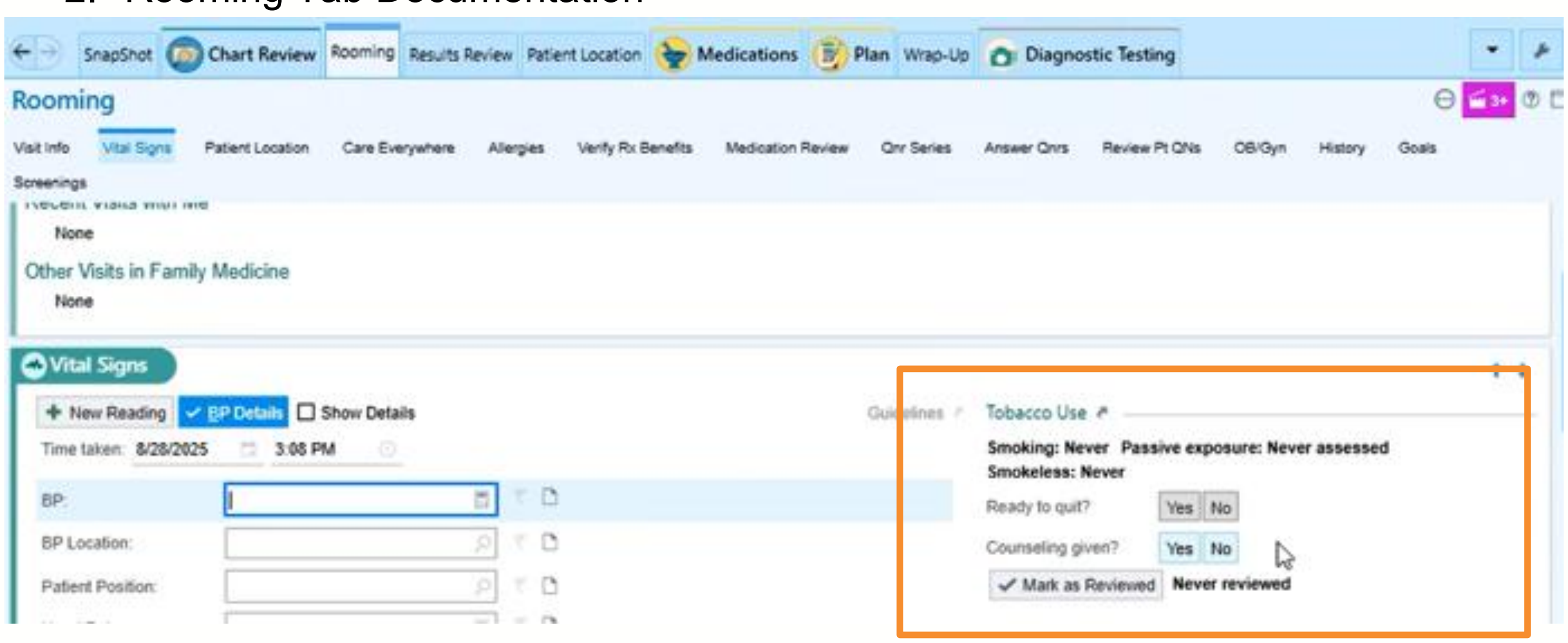

<u>Measures of Succes:</u>

1. Prior to implementation of advice workflow, Clinic Site Manager's should pull the Tobacco Cessation Report within EPIC dashboards to see their clinics baseline levels for providing/documenting tobacco cessation counseling
    a. Reports can be stratified by participant socio-demographics to identify current trends.
    b. Baseline report should then be compared to new reports to assess changes in tobacco cessation counseling delivery.

Measure	Description	Target Benchmark
**% of Tobacco Users Who Receive Advice to Quit**	Advice documented	≥ 80%
**Advice equity**	Review by socio-demographic and organizational factors such as age, race/ethnicity, language, site, etc.	Identify gaps

Assist

Who to Assist in Developing a Quit Plan:

For **patients willing to make a quit attempt**, assist with **creating a quit plan**, **offer medication**, and **provide or refer the patient for counseling to help them quit**.

- Evidence strongly suggests that the combination of medication and counseling dramatically improves the chances of quitting successfully.
- The action steps below may be completed by an individual clinician or by a patients care team.

How to Develop a Quit Plan:

1. As part of a patient's preparation for quitting, encourage them to take the following steps (STAR):
    a. **Set** and stick with your quit date, ideally within 2 to 4 weeks
    b. **Tell** your family, friends, and coworkers about your quit date. Request their understanding and support.
    c. **Anticipate** and prepare for challenges. Some examples are nicotine withdrawal symptoms, being around others who use tobacco.
    d. **Remove** tobacco products and paraphernalia from your environment. You're your home and vehicles tobacco-free.

For **adolescent/pediatric patients**, the US Preventative Task Force ***does not*** **recommend nicotine replacement therapy**. Patients aged 13-17 should discuss their behavioral quit plan and receive a **referral to Kick It CA** for cessation support.

Review Medication Options:

1. Explain how medication reduces withdrawal symptoms and makes it more likely that patients will succeed in quitting. Consider using a combination of therapy to provide patients with both long-acting and short-acting (craving relief) medication.

    a. FDA-approved medications[16,17] include the following:

        - **Short-acting (rescue from cravings):** Nicotine gum, nicotine lozenge, nicotine inhaler, nicotine nasal spray
        - **Long-acting:** Nicotine patch, bupropion SR (non-nicotine pill and nicotine antagonist), varenicline (non-nicotine pill, nicotine antagonist, and nicotine agonist), patch and bupropion SR in combination

Medication Type	Availability	Caution/Warning	Side Effects	Use
		**Short-Acting**		
**Nicotine Gum**	Over-the-Counter (OTC)	Use caution with dentures<br><br>Do not eat or drink 15 minutes before use or during use	Mouth soreness<br><br>Stomach ache	After quitting: Up to 12 weeks<br><br>*Optional* before quitting: Up to 6 months prior to quit date with smoking reduction
**Nicotine Nasal Spray**	Prescription (Rx)	Not for patients with asthma<br><br>May irritate nose<br><br>May cause dependence<br>Do not inhale	Nasal irritation	3–6 months, tapering at end
**Nicotine Inhaler**	(Rx)	May irritate mouth or throat at first	Local irritation of mouth and throat	After quitting: Up to 6 months, tapering at end<br><br>*Optional* before quitting: Up to 6 months prior to quit date with smoking reduction
**Nicotine Lozenge**	OTC	Do not eat or drink 15 minutes before use or during use<br><br>Take one lozenge at a time<br><br>Use no more than 20 in 24 hours	Hiccups<br>Cough<br>Heartburn	3–6 months
		**Long Acting**		
**Patch**	OTC	Do not use if you have severe eczema or psoriasis.	Local skin reaction<br><br>Insomnia	After quitting: 12 weeks<br><br>*Optional* before quitting: Up to 6 months prior to quit date with smoking

**Wellbutrin/Zyban (Bupropion)**	(Rx)	Not for use if you:<br><br>Use monoamine oxidase inhibitors<br>Use other forms of bupropion<br><br>Have a history of seizures<br><br>Have a history of eating disorders	Insomnia<br><br>Dry mouth	Start 1–2 weeks before quit date<br><br>Use for 2–6 months
**Chantix (Varenicline)**	(Rx)	Use with caution if you:<br><br>Have renal impairment<br><br>Have serious psychiatric illness<br><br>Are undergoing dialysis	Nausea<br><br>Insomnia<br><br>Abnormal dreams	Start 1 week before quit date<br><br>Use for 3–6 months
**Combinations**<br><br>• Nicotine patch + gum/bupropion<br><br>• Nicotine gum or lozenge + patch or varenicline<br><br>• Bupropion + nicotine medication or varenicline<br><br>• Varenicline + gum/lozenges and/or bupropion	(Rx)	Only patch plus bupropion is FDA-approved.	See Above	See Above

Documentation Guidance:

1. *Tobacco Cessation ExpressLane for Providers*: Refer to SYH's tobacco express lane to learn how to automatically document counseling treatment plan and Kick It CA referral.
    a. For **pediatric/adolescent patients**
        - Ensure all documentation is completed with the confidential note

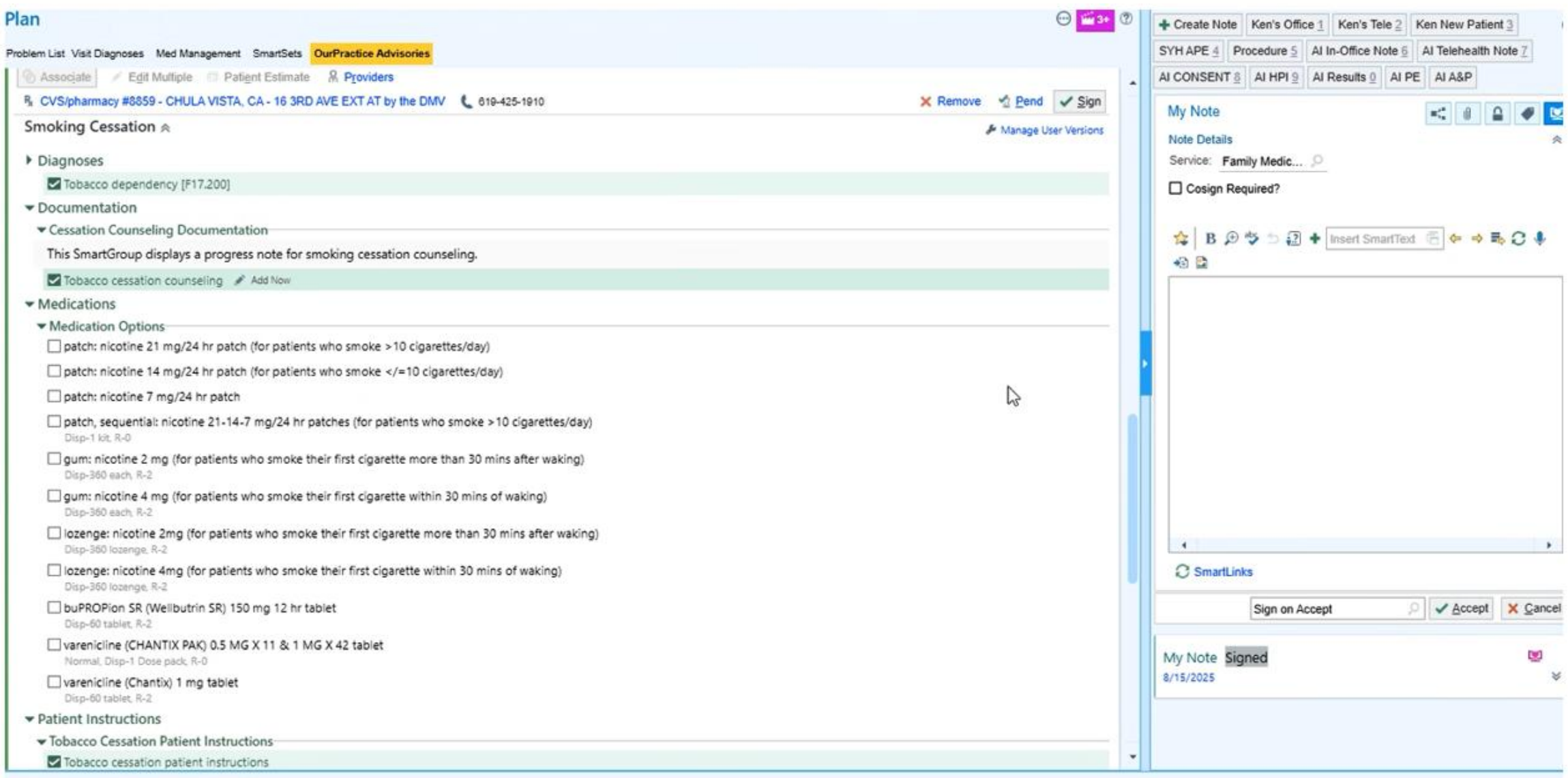

Measures of Success:

1. Prior to implementation of assist workflow, Clinic Site Manager's should pull the Tobacco Cessation Report within EPIC dashboards to see their clinics baseline referral rate for tobacco/nicotine treatment.
    a. Reports can be stratified by participant socio-demographics to identify current trends.
    b. Baseline report should then be compared to new reports to assess changes in motivational readiness

Measure	What It Evaluates	Suggested Target
**Cessation Medication Initiation**	% of eligible patients prescribed NRT or other treatment plan	≥ 50% of tobacco users

Other Considerations:

1. Consider using open "pocket" prescriptions for nicotine patches to ensure timely intervention.

Arrange

Who to Arrange Follow-Ups For:

All patients with a quit plan should receive follow-up to monitor challenges and medication adherence. Follow-up can take place at a **clinical appointment or via telehealth.**

- For **pediatric/adolescent patients** a 2-week follow-up should occur with a Health Educator or during a confidential visit.

How to Schedule Follow-Up Appointments:

1. Schedule follow-up with Health Educator and/or RN ideally within 2 weeks of quit date or medication start.
2. Use RN medication reconciliation appointment slots for quicker access when PCP availability is limited.
    a. RN/HE should inform PCP of any challenges or changes needed to treatment
3. Follow-up appointments with Health Educators should be scheduled to continue providing ongoing support during initial stages of quitting

Documentation Guidance:

- Confirm appointment creation in EPIC
    a. Date
    b. Time
    c. Who appointment is scheduled with
- Note data of patient's selected quit date

Ordering CT Scan for Lung Cancer Screening:

1. MA can pend an order for a CT scan in EPIC after screening procedures
2. PCP can place an order for CT scan after examination.
    1. PCP can also approve pending orders created by MA
3. Linkage to Referrals Specialist should be made for patients who have not completed their CT scan or express need for support.

Measures of Success:

Measure	What It Evaluates	Suggested Target

**Scheduled Appointments**	% of patients with scheduled follow-up within 2 weeks	≥ 75%
**Follow-Up Completion Rate**	% of scheduled patients that were kept, no-show, or cancelled	Used to identify trends in appointment outcomes

💡 Site-Level Considerations for Clinic Managers 💡

Clinic managers play a key role in building awareness, aligning staffing, and integrating tobacco cessation into team workflows. Below are high-impact strategies to reinforce smoking cessation efforts across SYHealth sites.

1. **Quality Improvement & Data Review**
   - Utilize the Tobacco Cessation Report to review trends in tobacco cessation efforts within Continuous Quality Improvement meetings
   - Utilize Kick It CA referral report to identify trends related to external referrals for tobacco cessation.

Focus Area	Action Item	Measures of Success
**Tobacco Cessation Quality Scorecard**	Coordinate with Quality Department to integrate tobacco cessation quality measures into scorecard	• Quality scorecard includes tobacco cessation measures
**Monthly CQI Review**	Clinic managers should include a review of key tobacco cessation performance quality measures during monthly CQI meetings. This includes:<br>• Tobacco use screening rate<br>• Patients motivational readiness<br>• Cessation counseling documentation<br>• Medication or treatment initiation<br>• Kick It California referral and engagement reports<br>Use these data to identify workflow gaps, celebrate successes, and conduct PDSA cycles.	• ≥ 95% of patient screened<br>• ≥ 95% of smokers have documented motivational readiness<br>• ≥ 95% of smokers received tobacco cessation counseling<br>• ≥ 50% of tobacco users who discussed a quit plan are prescribed treatment plan<br>• ≥ 95% of smokers are provided with Kick It CA referral

2. **Outreach & Awareness**

Strategy	Action Items	Measures of Success
**Tobacco Cessation Month**	Leverage November for targeted outreach. Promote the Great American Smokeout (3rd Thursday) and Lung Cancer Awareness Month to highlight tobacco-related health risks and encourage quit attempts.	• Type of events<br>• # of events<br>• # of patients reached
**Flyers**	Post educational materials in waiting rooms, exam rooms, and health education areas.<br><br>*ensure materials are culturally tailored and relevant to patient populations<br><br>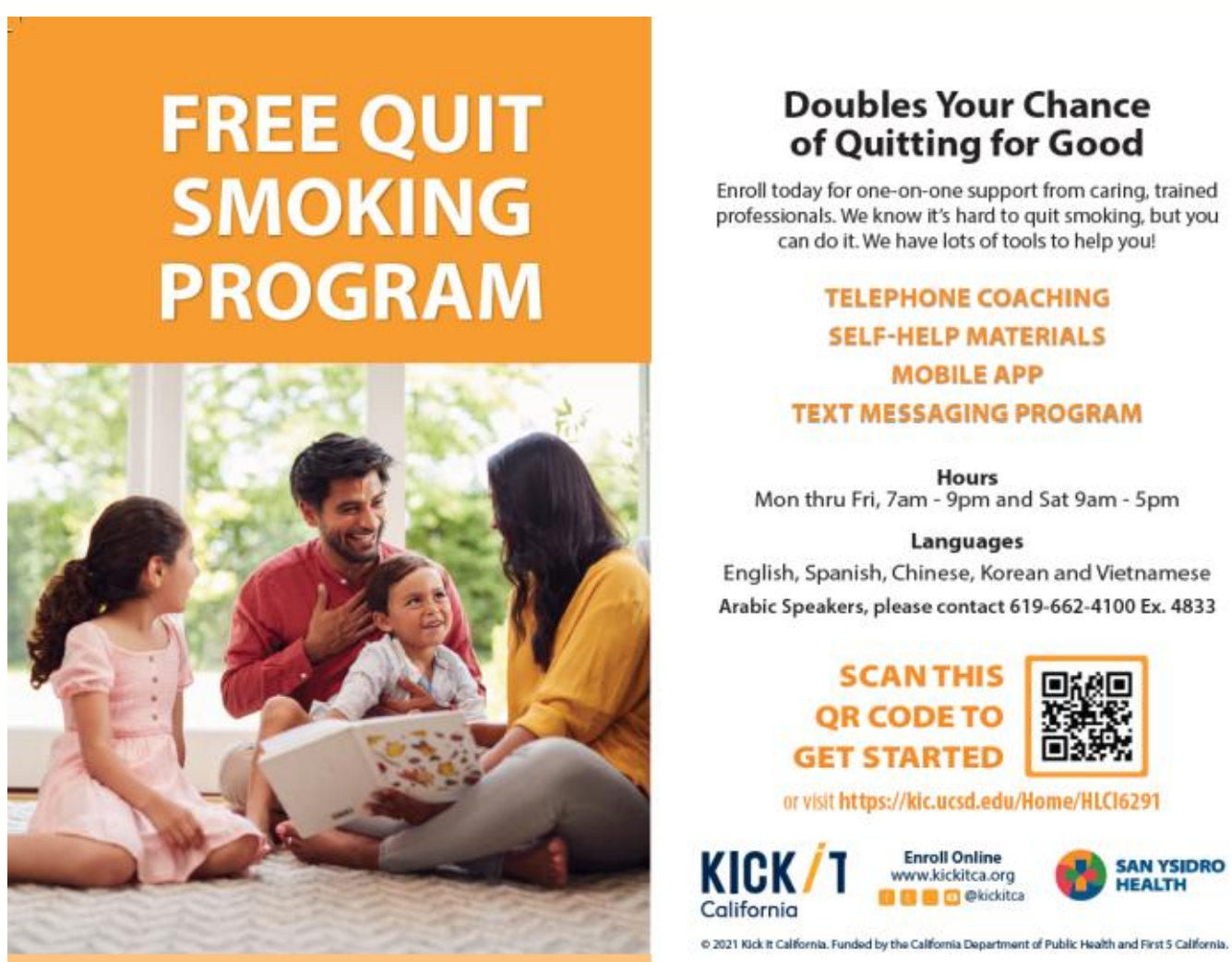	• # of clinics utilizing poster<br>• # of flyers posted

	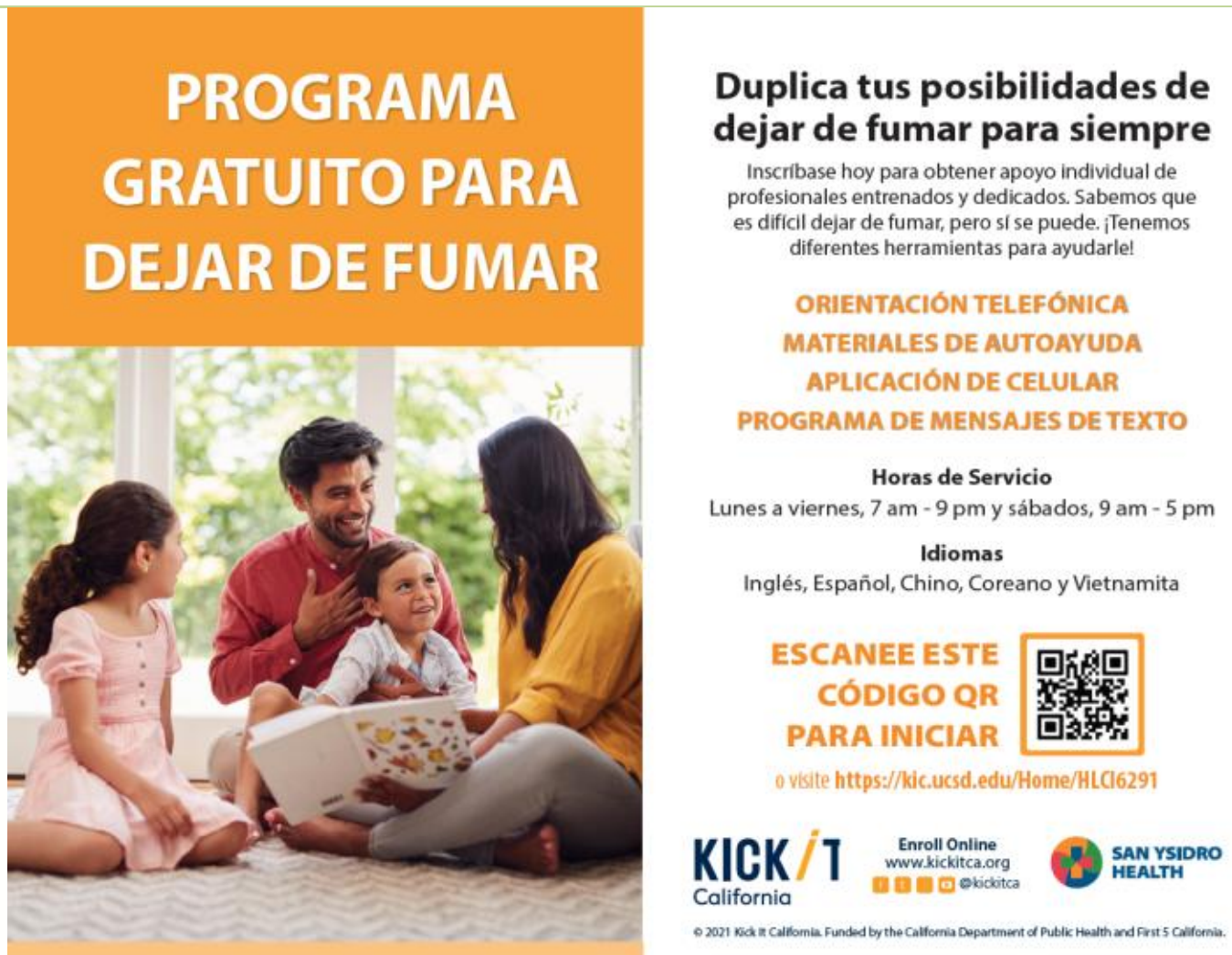	
**TV Messaging**	Circulate looping visuals and animated posters on clinic TVs highlighting health risks, medication options, and how to get support.	• # of tobacco cessation related videos circulating
**Tabling Events**	Incorporate cessation messaging into existing outreach events like Super Saturday. Offer mini counseling sessions or live demonstrations on NRT tools. Use these events to generate referrals for tobacco cessation counseling	• # of Super Saturday events participated<br>• # of participants reached
**Tobacco Cessation Support Groups**	Support group of patients quitting tobacco use	• # of participants reached<br>• # of sessions conducted

3. Staffing & Capacity

Need	Recommended Action	Measures of Success
**Hire Dedicated Tobacco Cessation Counselors**	Hire certified tobacco cessation counselors to support motivational interviewing, treatment planning, and follow-up.	• # (or %FTE) of new staff hired for tobacco cessation
**Expand Existing Roles**	Upskill 1-2 existing staff per clinic site to support with tobacco cessation efforts.	• # (or %FTE) of existing staff with an expanded tobacco cessation role

**Cross-Training**	Explore cross-training existing staff for backup coverage and patient continuity.	• # (or %FTE) staff cross-trained
**Training**	Conduct periodic training to review tobacco cessation workflows, documentation procedures with clinical staff	• # of training conducted
**Communication Pools**	Create communication pool specifically for tobacco cessation to ensure that PCP/RN/MA is in constant communication regarding patient needs/prescriptions/follow ups	• # of communication pools created

4. **Cross Departmental Integration**

Department	Integration Opportunities	Measures of Success
**Dental Services**	Implement brief tobacco screenings during oral exams. Refer patients to Health Educator or PCPs.	• # of referrals to primary care for tobacco cessation for motivated patients
**Social Services**	Provide tobacco cessation information to social services who can refer patients to Kick It CA or Health Education	• # of informational brochures/flyers provided to social services<br>• # referrals made to Kick It CA<br>• # of referrals to Health Education for tobacco cessation
**Referrals Department**	Connect with referral department to identify best practices in following up with patients referred to for lung cancer screening	• # of patients referrals department supports to complete lung cancer screening
**All Staff Meetings**	Join periodic All Staff Meetings to share tobacco cessation workflow and	• # of All Staff Meeting presentations

	resources. All staff who have the potential of interacting with patients should have basic knowledge to be able to assist patients if needed	

Tobacco Cessation Counselor Job Description - Example

Department:	Location:	Supervisor Title:
Job Code:	Budget Code:	Job Status: Exempt ☐ Non-Exempt ☒
Subordinate Title(s):		

Position Summary: Under the direction of the **[Program Manager],** the Tobacco Cessation Counselor is responsible for providing individual and group tobacco cessation counseling to the patients we serve. The Tobacco Cessation Counselor works with patients to support the reduction of tobacco and nicotine product use through motivational interviewing techniques. The Tobacco Cessation Counselor is well trained in all Nicotine Replacement Therapies to help develop a preliminary quit plan with the patient. The Tobacco Cessation Counselor works hand-in-hand with the clinical team to ensure treatment is accessible and offers follow-up support to patients.

Essential Functions of the Job:

1. Provides individual and group tobacco cessation counseling to patients who screen positive for tobacco and nicotine use to help patients overcome nicotine addiction.
2. Creates personalized plans that incorporate motivational interviewing, behavioral techniques, and medication when appropriate
3. Evaluates the patient's specific situation, including tobacco use patterns, readiness to quit, and any co-occurring conditions.
4. Connects individuals with additional resources, such as support groups, medication, providers, or other healthcare professionals.
5. Monitors client progress, documenting session details, and adjusting strategies as needed.
6. Keeps abreast of the latest research, guidelines and best practices in tobacco cessation.
7. Meets productivity expectation.
8. Proficiently maneuvers through all electronic applications used for job.
9. Performs other job-related duties as needed, assigned and/or as requested.
10. Adheres to SYHealth policies and best practices, ensuring staff compliance to attendance and punctuality.

Additional Duties and Responsibilities:

1. Enhances professional growth and development through participation in educational programs.
2. Attends meetings as required and participates in committees as directed. Adheres to all applicable SYHealth policies, procedures, protocols, and best practices.

3. Demonstrates effective verbal and written communication skills in collaboration with teams to achieve quality, excellence, harmony and problem-solving to obtain stated goals and objectives.
4. Demonstrates active listening skills, respect and a positive “Can Do” attitude.
5. Demonstrates professional behavior standard etiquette, courtesy, mutual respect, confidentiality and treats every patient and employee with dignity in every interaction.
6. Exhibits an elevated level of integrity, ethics, transparency, leadership style and trust.
7. Promotes and fosters respect for patient privacy by maintaining confidentiality on all patient EHR data and abides by both the HIPAA Privacy and Security rules.
8. Exhibits excellent presentation skills, confidently speaks up, follows the appropriate chain of command
9. Maintains a clean, safe, and organized work area always.
10. Demonstrates supportive skills in providing excellent customer service experiences

Job Requirements:

Education Required (Minimum level of education): Bachelor’s Degree in public health, social services, psychology or related field and/or equivalent experience.

Certifications/Licenses Required:

1. CA driver’s license with appropriate insurance.
2. Tobacco Treatment Specialist (CTTS) Certification or completion of a similar program (or willing to obtain within 30 days of hire)
3. Human Subjects Protection Certificate (or willing to obtain within 30 days of hire)

Experience Required (minimum level of experience): Has experience in direct customer service, motivational interviewing, health education or counseling.

Preferred: One year experience in a providing tobacco cessation or substance use disorder counseling services

Verbal and Written Skills Required to Perform the Job:

1. Excellent oral and written communication skills.
2. Provides positive customer service skills.
3. Is an active listener and provides support and guidance to others.
4. Bilingual (English/Spanish)

Technical Knowledge and Skills Required to Perform the Job: Knowledge of tobacco treatment modalities. Demonstrate ability to be culturally sensitive and appropriate in working with minority communities. Demonstrate respect for diversity. Exercise tact, objectivity, sensitivity, strategy, and judgment in dealing with patients. Organize, set priorities, and exercise sound independent judgment within areas of responsibility. Establish and maintain effective working relationships patients, other professional staff, and the public.

Equipment Used Computers, Software systems, Scanner, Telephones, Copy and Fax machines. Microsoft Suite, Teams, and other database management software (ex: REDCap)

Working Conditions and Physical Requirements

- Prolonged, extensive, and considerable standing/sitting/walking/lifting. Manual dexterity and mobility.
- Always reaching, stooping bending, kneeling, crouching.
- Good organizational skills and ability to remain focused and concentrate with noise around.
- Ability to handle multiple job task functions simultaneously. Ability to work harmoniously with others as a team member.
- May be required to work evenings and/or weekends, and provide staffing coverage at any SYHealth facility as needed.

Universal Requirements: Pre employment requirements include I-9, physical, positive background and reference check results, complete application, new hire orientation, pre employment PPDs.

HIPAA/Compliance

References

1. Centers for Disease Control and Prevention (CDC). Diseases and Death. 2022. Accessed December 23, 2023. https://www.cdc.gov/tobacco/data_statistics/fact_sheets/fast_facts/diseases-and-death.html

2. Cornelius ME, Loretan CG, Wang TW, Jamal A, Homa DM. Tobacco Product Use Among Adults — United States, 2020. *MMWR Recommendations and Reports*. 2022;71(11):397-405. doi:10.15585/mmwr.mm7111a1

3. Lin SC, Gathua N, Thompson C, Sripipatana A, Makaroff L. Disparities in smoking prevalence and associations with mental health and substance use disorders in underserved communities across the United States. *Cancer*. 2022;128(9):1826-1831. doi:10.1002/cncr.34132

4. Centers for Disease Control and Prevention. Smoking is down, but almost 38 million American adults still smoke . 2018. Accessed December 23, 2023. https://archive.cdc.gov/www_cdc_gov/media/releases/2018/p0118-smoking-rates-declining.html

5. Flocke SA, Hoffman R, Eberth JM, et al. The prevalence of tobacco use at federally qualified health centers in the United States, 2013. *Prev Chronic Dis*. 2017;14(4):1-6. doi:10.5888/pcd14.160510

6. American Lung Association. Tobacco Cessation Coverage and Federally Qualified Health Centers. Published online 2020.

7. U.S. Department of Health and Human Services. *Smoking Cessation: A Report of the Surgeon General*.; 2020. doi:10.1097/JU.0000000000001114

8. Tan ASL, Young-Wolff KC, Carter-Harris L, Salloum RG, Banerjee SC. Disparities in the receipt of tobacco treatment counseling within the US context of the affordable care act and meaningful use implementation. *Nicotine and Tobacco Research*. 2018;20(12):1474-1480. doi:10.1093/ntr/ntx233

9. Siddiqi AD, Britton M, Chen TA, et al. Tobacco Screening Practices and Perceived Barriers to Offering Tobacco Cessation Services among Texas Health Care Centers Providing Behavioral Health Treatment. *Int J Environ Res Public Health*. 2022;19(15):1-15. doi:10.3390/ijerph19159647

10. Geletko KW, Graves K, Lateef H, Harman J. Tobacco Cessation Counseling and Medications Provided by Physicians to Tobacco Users During Primary Care Visits. *J Prim Care Community Health*. 2022;13. doi:10.1177/21501319221093115

11. Dai H, Clements M. Trends in Healthcare Provider Advice on Youth Tobacco Use, 2011–2015. *Am J Prev Med*. 2018;55(2):222-230. doi:10.1016/j.amepre.2018.04.015

12. Allen CG, Cotter MM, Smith RA, Watson L. Successes and challenges of implementing a lung cancer screening program in federally qualified health centers: a qualitative analysis using the Consolidated Framework for Implementation Research. *Transl Behav Med*. 2021;11(5):1088-1098. doi:10.1093/tbm/ibaa121

13. National Committee for Quality Assurance. Medical Assistance with Smoking and Tobacco Use Cessation. 2021. Accessed January 29, 2024. https://www.ncqa.org/hedis/measures/medical-assistance-with-smoking-and-tobacco-use-cessation/

14. United States Preventive Services Taskforce. Information for Professionals . Accessed July 18, 2025. https://www.uspreventiveservicestaskforce.org/uspstf/recommendation-topics/tools-and-resources-for-better-preventive-care

15. Fiore MC, Jaen Roberto C, Baker TB, et al. Treating tobacco use and dependence: 2008 update. *Clinical Treatment Guide For Tobacco Use and Dependence*. 2008;(May):1-187.

16. Food and Drug Administration. Smoking - Medicines To Help You Quit. Accessed July 19, 2025. https://www.fda.gov/consumers/womens-health-topics/smoking-medicines-help-you-quit#Other

17. Centers for Disease Control and Prevention. Clinical Cessation Tools. Accessed July 19, 2025. https://www.cdc.gov/tobacco/hcp/patient-care/clinical-cessation-tools.html
